# Supplementary material for: Synthesis and Anti-Leishmanial Properties of Quinolones Derived from Zanthosimuline
Source: Molecules. 2022 Nov 15;27(22):7892. doi: 10.3390/molecules27227892 (PMC9693141; doi:10.3390/molecules27227892)
Supplement: Supplementary file 1 [file molecules-27-07892-s001.zip › molecules-1957414-supplementary.pdf]

Synthesis and Anti-Leishmanial Properties of Quinolone Derived from Zanthosimuline

Gwenaëlle Jézéquel [1], Laura Nogueira de Faria Cardoso [1], Florent Olivon [1], Indira Dennemont [2], Cécile Apel [1], Marc Litaudon [1], Fanny Roussi [1], Sébastien Pomel [2] and Sandy Desrat [1,*]

1 Université Paris-Saclay, CNRS, Institut de Chimie des Substances Naturelles, UPR 2301, 91198 Gif-sur-Yvette, France
2 CNRS, BioCIS, Université Paris-Saclay, 92290 Châtenay-Malabry, France
* Correspondence: sandy.desrat@cnrs.fr

Supporting Information

NMR & HRMS spectra of the compounds

7 ^{1}H NMR $CDCl_3$ 500MHz

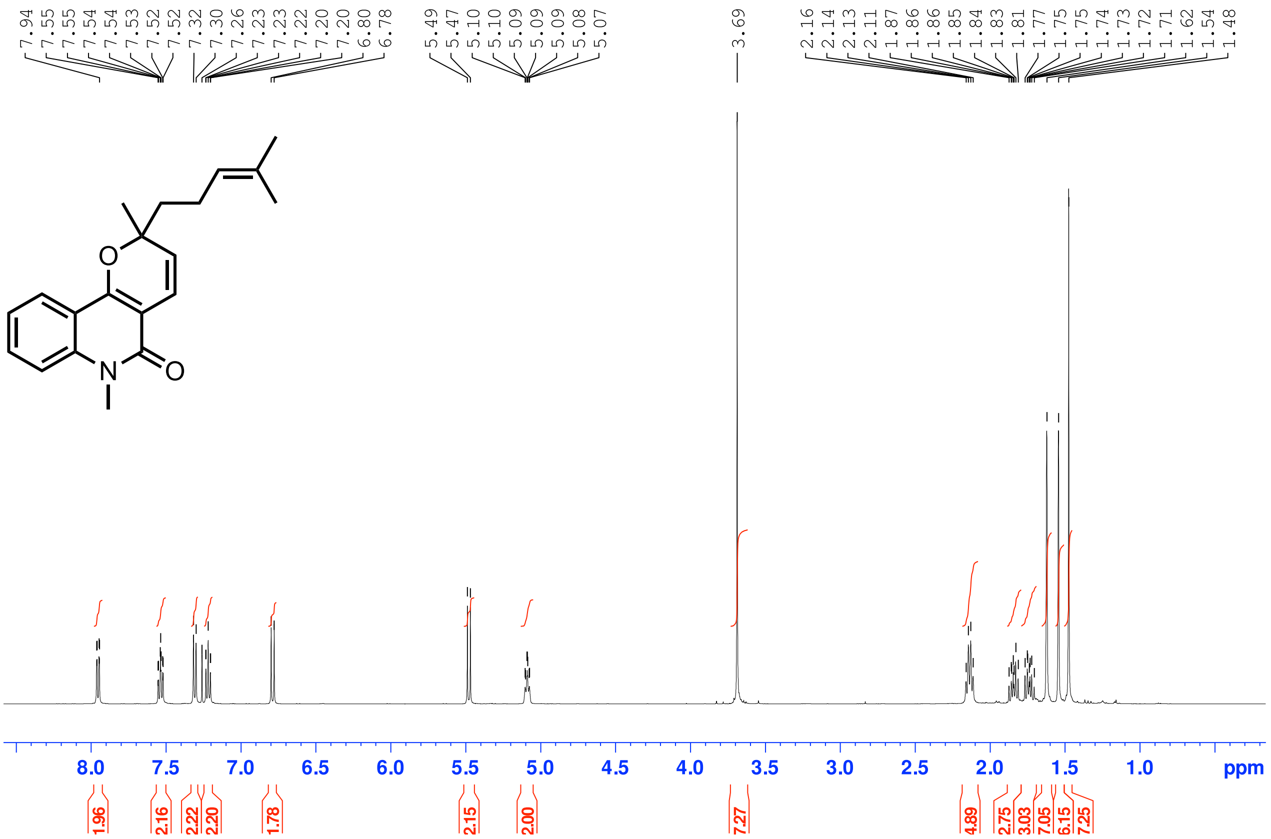

7 ^{13}C NMR $CDCl_3$ 125MHz

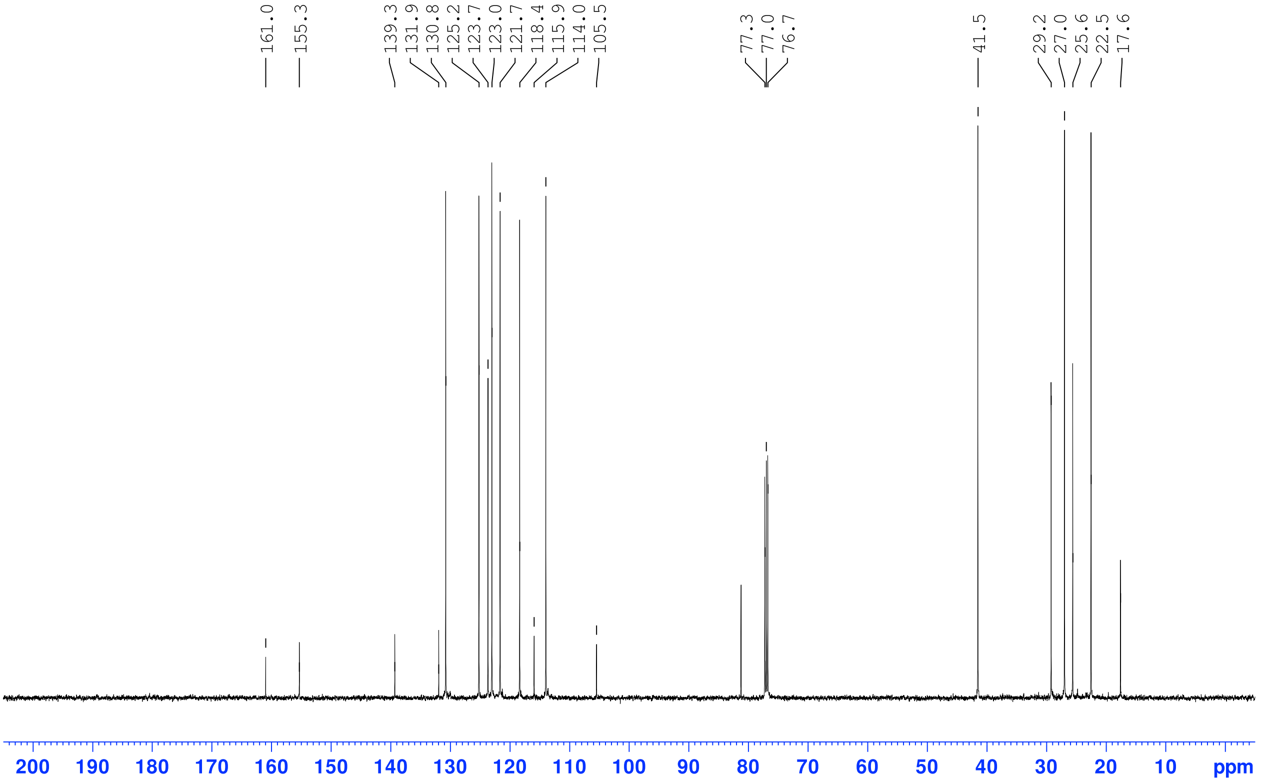

7 HRMS (ESI+)

Single Mass Analysis
Tolerance = 5.0 PPM / DBE: min = -1.5, max = 100.0
Element prediction: Off
Number of isotope peaks used for i-FIT = 9

Monoisotopic Mass, Even Electron Ions
265 formula(e) evaluated with 1 results within limits (all results (up to 1000) for each mass)
Elements Used:
C: 0-100 H: 0-120 N: 0-6 O: 0-10

03-Jul-2017 15:52:36 LCT Premier XE KE483 GUERITTE_desrat45-1 655 (3.059)
1: TOF MS ES+

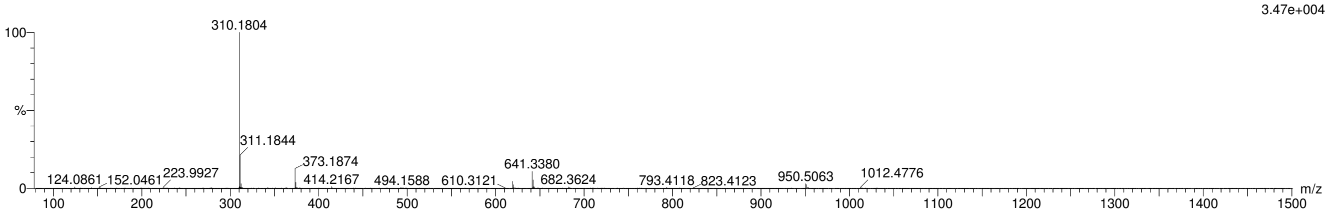

Minimum:				-1.5			
Maximum:		5.0	5.0	100.0			

Mass	Calc. Mass	mDa	PPM	DBE	i-FIT	i-FIT (Norm)	Formula
310.1804	310.1807	-0.3	-1.0	9.5	629.0	0.0	C20 H24 N O2

8 ^{1}H NMR $CDCl_3$ 500MHz

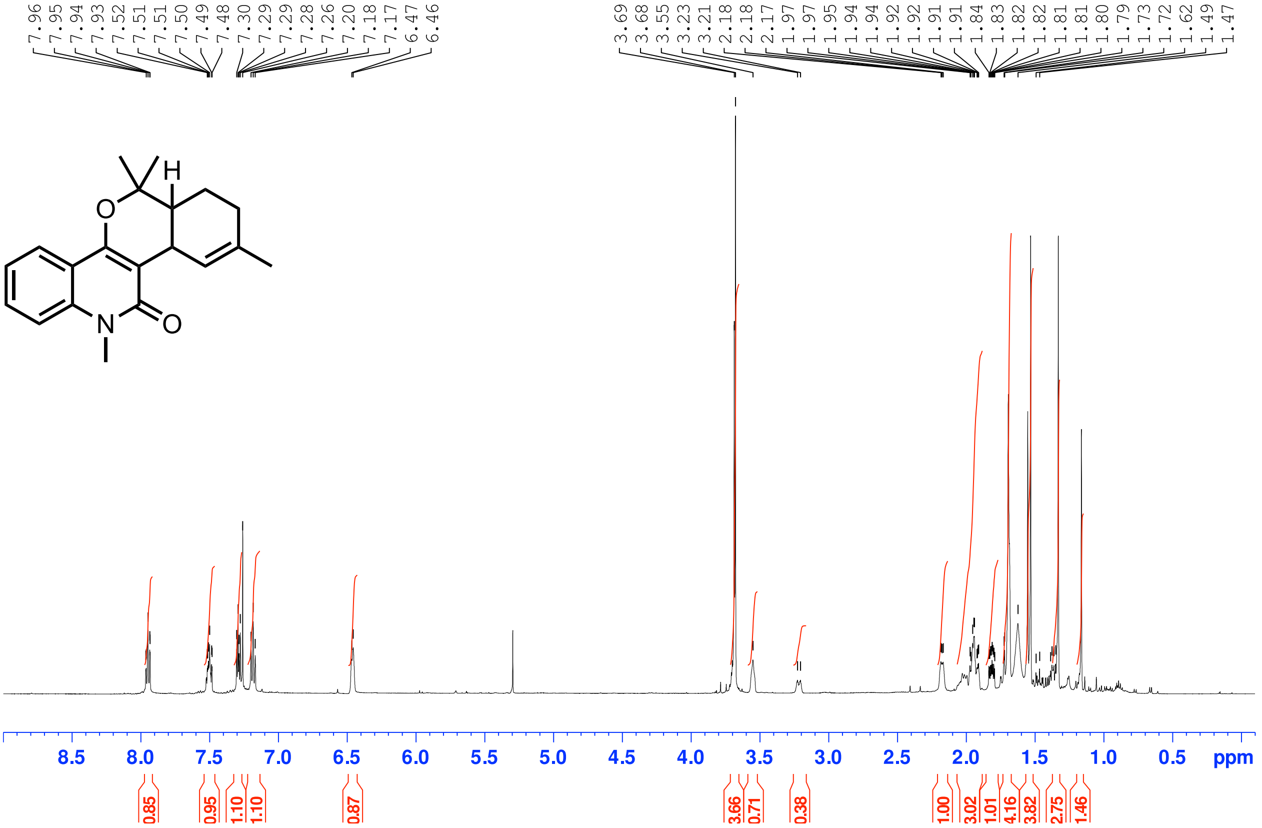

8 HRMS (ESI+)

Single Mass Analysis
Tolerance = 5.0 PPM / DBE: min = -1.5, max = 100.0
Element prediction: Off
Number of isotope peaks used for i-FIT = 9

Monoisotopic Mass, Even Electron Ions
265 formula(e) evaluated with 1 results within limits (all results (up to 1000) for each mass)
Elements Used:
C: 0-100 H: 0-120 N: 0-6 O: 0-10
19-Jul-2017 15:19:42 LCT Premier XE KE483 GUERITTE_desrat57-1 665 (3.103)
1: TOF MS ES+

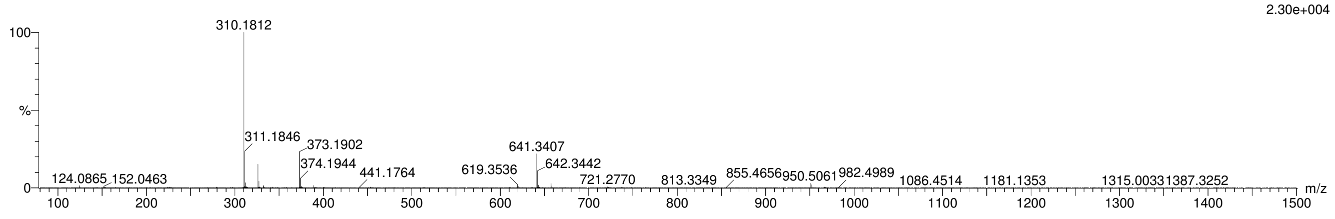

Minimum:				-1.5			
Maximum:		5.0	5.0	100.0			
Mass	Calc. Mass	mDa	PPM	DBE	i-FIT	i-FIT (Norm)	Formula
310.1812	310.1807	0.5	1.6	9.5	568.9	0.0	C20 H24 N O2

3 ^{1}H NMR $CDCl_3$ 500MHz

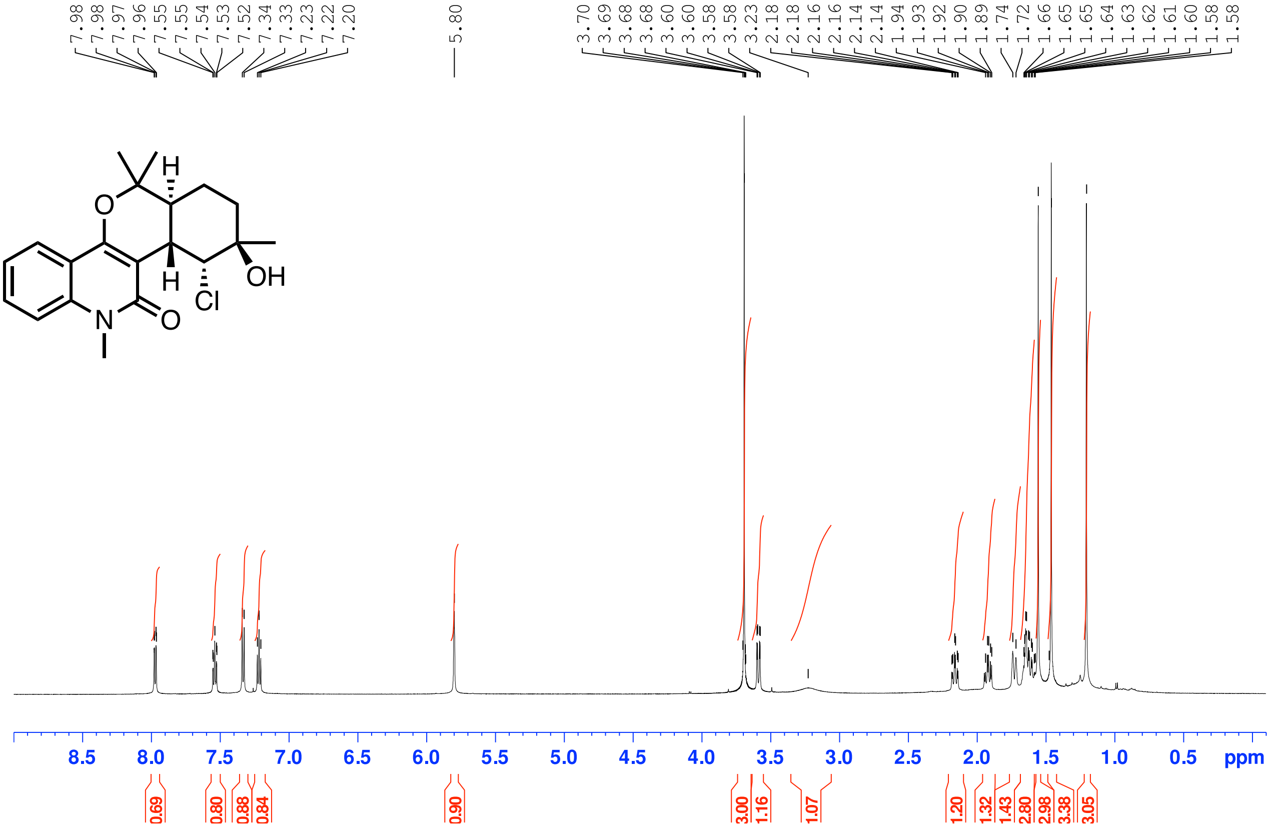

3 ^{13}C NMR $CDCl_3$ 125MHz

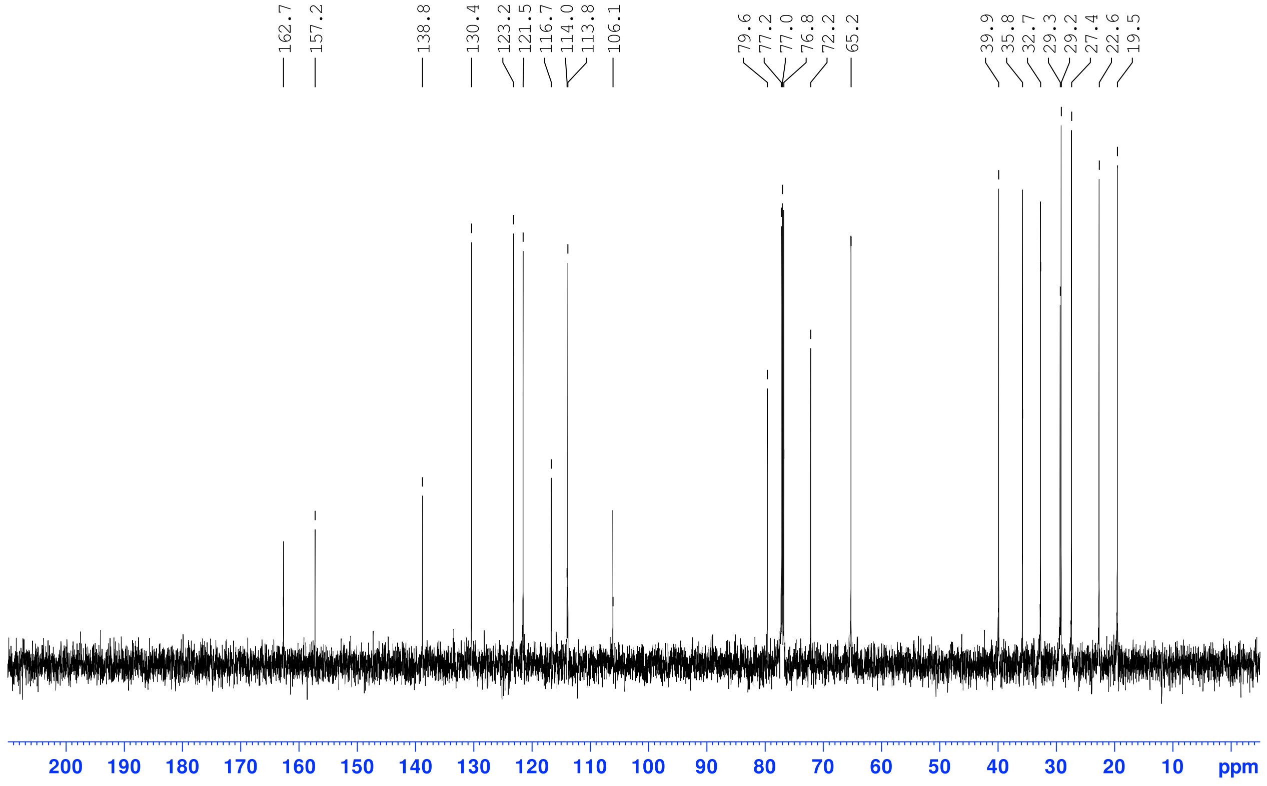

3 HRMS (ESI+)

Single Mass Analysis
Tolerance = 5.0 PPM / DBE: min = -1.5, max = 100.0
Element prediction: Off
Number of isotope peaks used for i-FIT = 9

Monoisotopic Mass, Even Electron Ions
508 formula(e) evaluated with 1 results within limits (all results (up to 1000) for each mass)
Elements Used:
C: 1-100 H: 0-100 N: 0-10 O: 0-16 Cl: 1-1

21-Sep-2017 17:54:59 LCT Premier XE KE483 GUERITTE_olivon159-3 567 (2.646)
1: TOF MS ES+

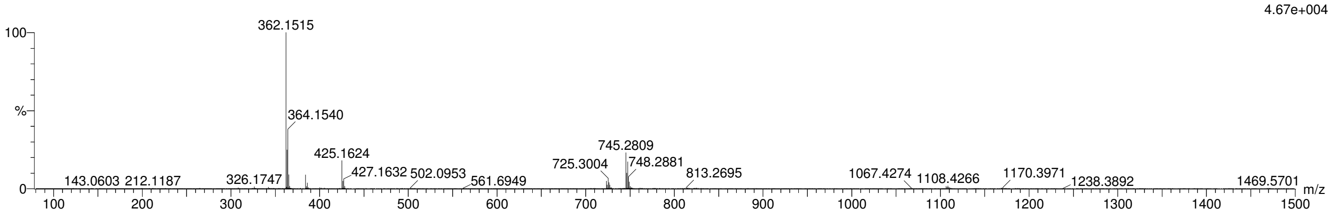

	Mass	Calc. Mass	mDa	PPM	DBE	i-FIT	i-FIT (Norm)	Formula
Minimum:					-1.5			
Maximum:			5.0	5.0	100.0			
	362.1515	362.1523	-0.8	-2.2	8.5	788.3	0.0	C20 H25 N O3 Cl

10 ^{1}H NMR CD_3CN 500MHz

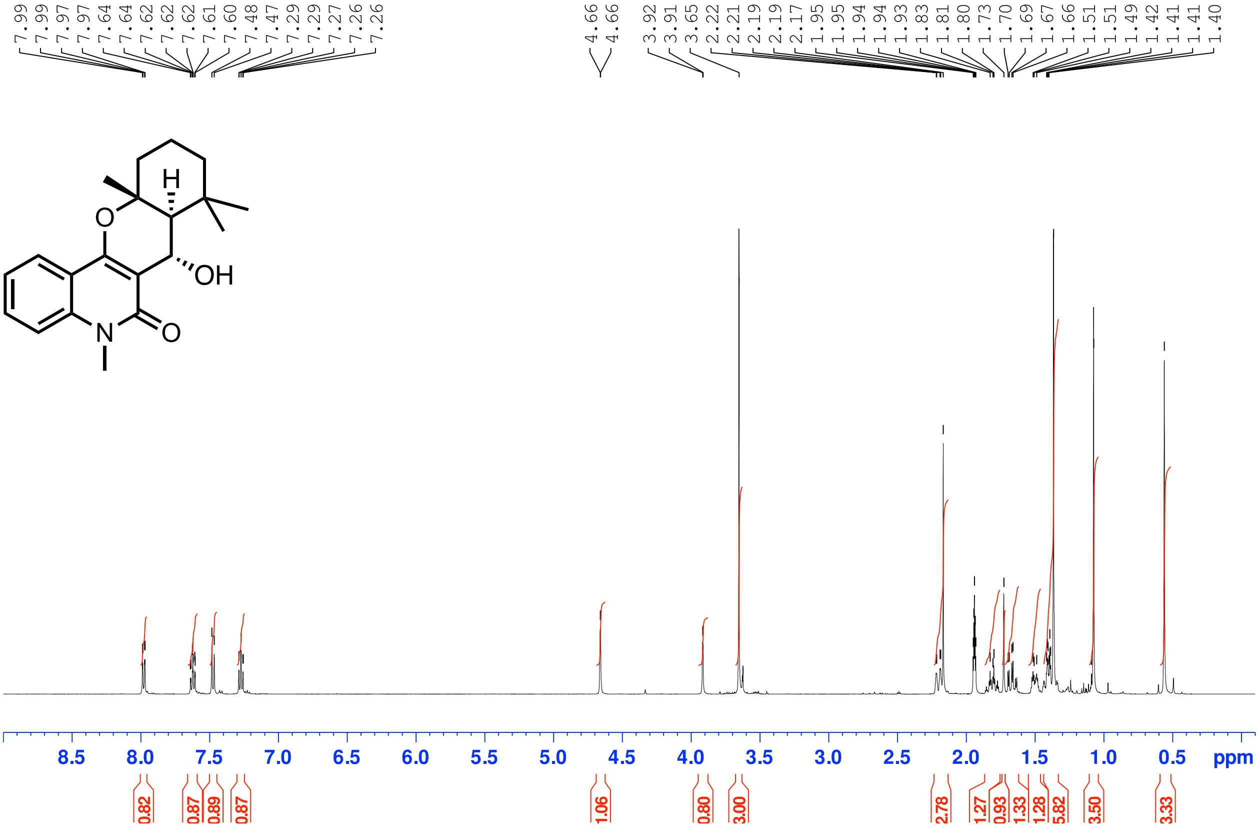

10 ^{13}C NMR CD_3CN 125MHz

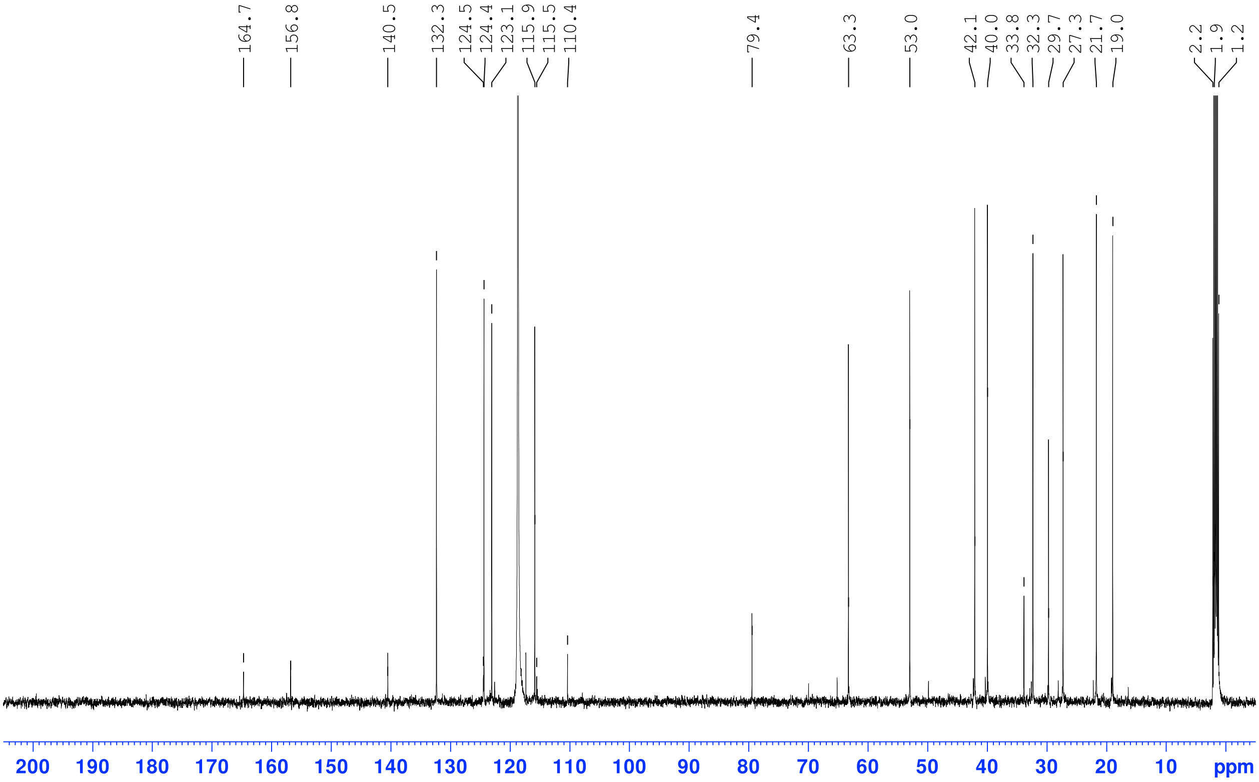

10 HRMS (ESI+)

Single Mass Analysis
Tolerance = 5.0 PPM / DBE: min = -1.5, max = 100.0
Element prediction: Off
Number of isotope peaks used for i-FIT = 9

Monoisotopic Mass, Even Electron Ions
265 formula(e) evaluated with 1 results within limits (all results (up to 1000) for each mass)
Elements Used:
C: 0-100 H: 0-120 N: 0-6 O: 0-10

03-Jul-2017 16:15:11 LCT Premier XE KE483 GUERITTE_desrat45-3 584 (2.758)
1: TOF MS ES+

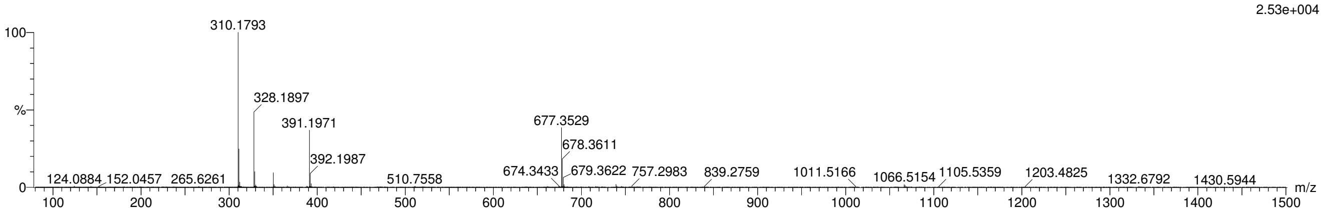

Minimum:				-1.5			
Maximum:		5.0	5.0	100.0			
Mass	Calc. Mass	mDa	PPM	DBE	i-FIT	i-FIT (Norm)	Formula
310.1793	310.1807	-1.4	-4.5	9.5	638.6	0.0	C20 H24 N O2

11 ^{1}H NMR $CDCl_3$ 500MHz

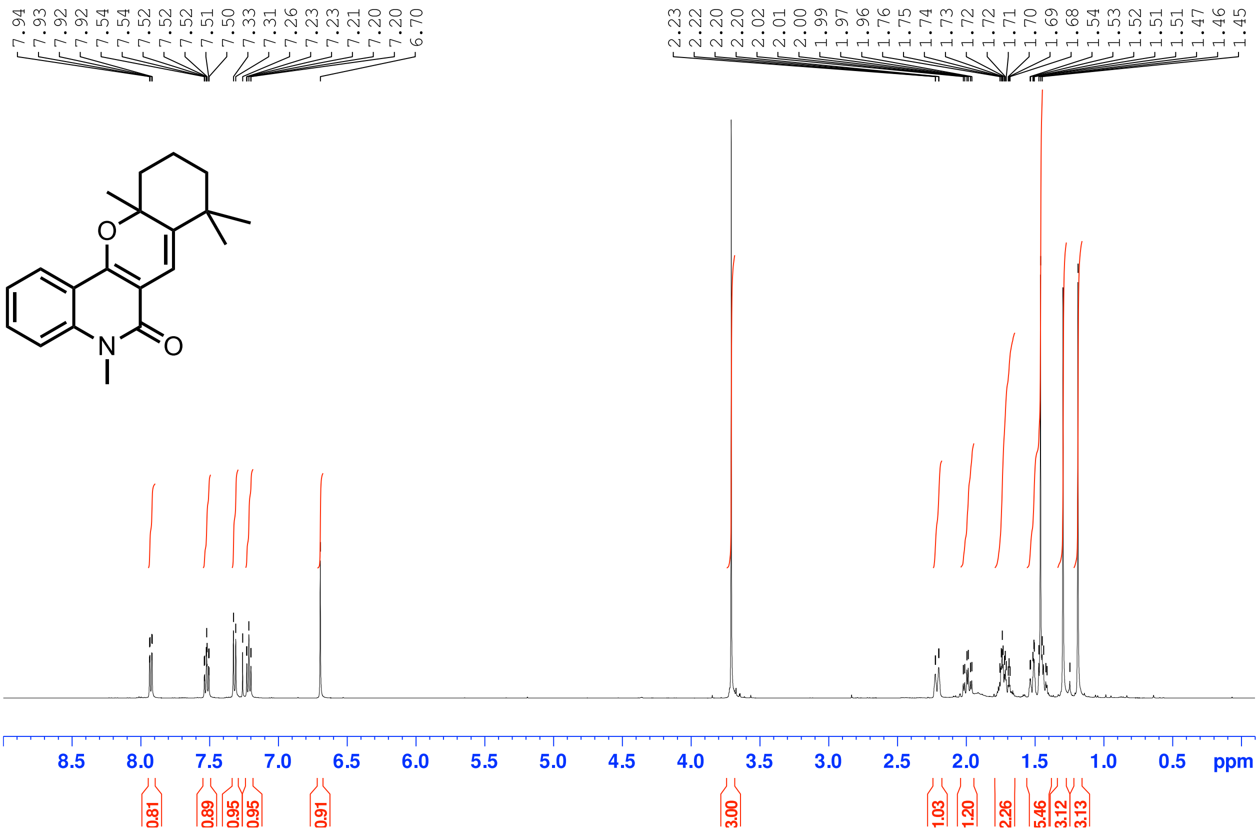

11 ^{13}C NMR $CDCl_3$ 125MHz

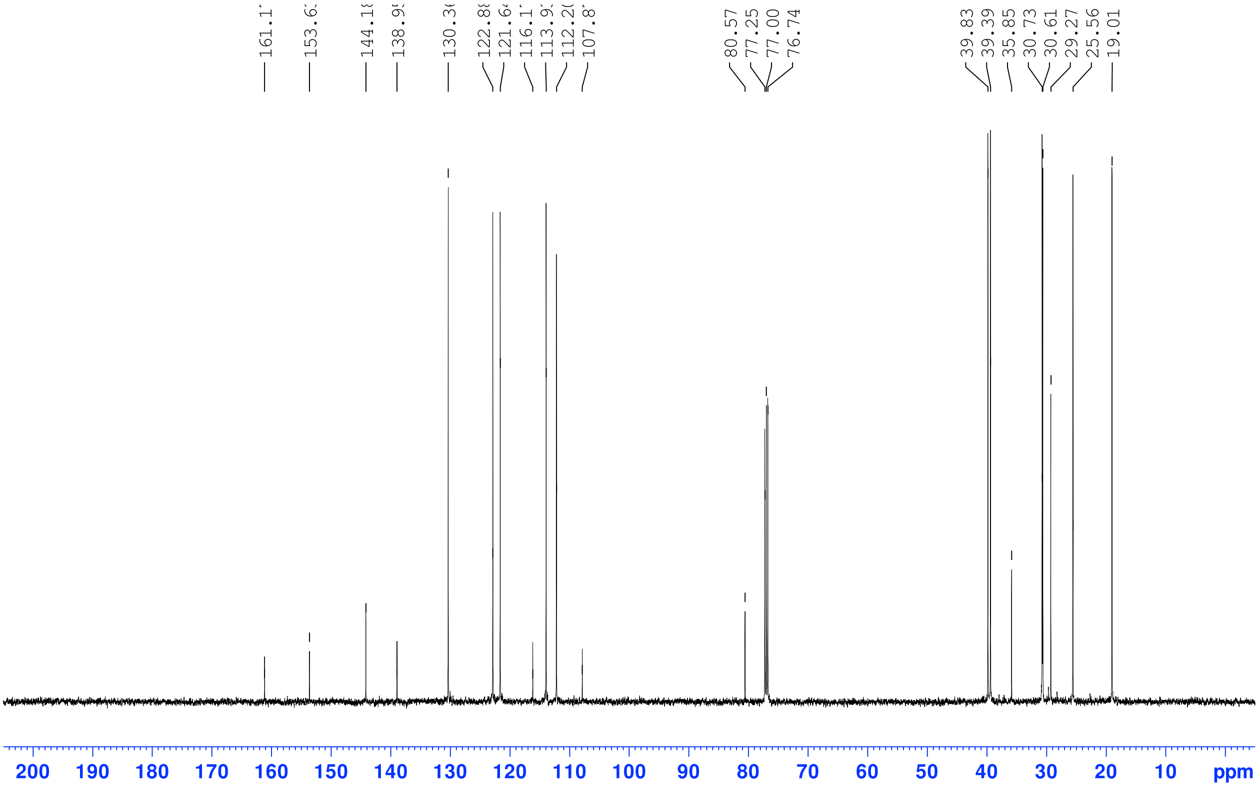

11 HRMS (ESI+)

Single Mass Analysis
Tolerance = 5.0 PPM / DBE: min = -1.5, max = 100.0
Element prediction: Off
Number of isotope peaks used for i-FIT = 9

Monoisotopic Mass, Even Electron Ions
265 formula(e) evaluated with 1 results within limits (all results (up to 1000) for each mass)
Elements Used:
C: 0-100 H: 0-120 N: 0-6 O: 0-10

03-Jul-2017 16:03:54 LCT Premier XE KE483 GUERITTE_desrat45-2 642 (3.017)
1: TOF MS ES+

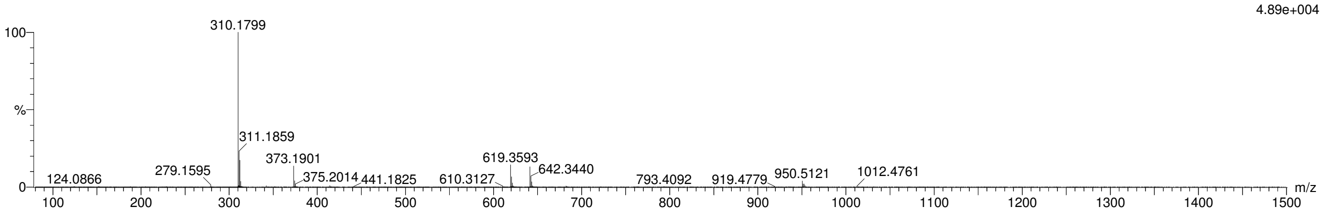

	Mass	Calc. Mass	mDa	PPM	DBE	i-FIT	i-FIT (Norm)	Formula
Minimum:					-1.5			
Maximum:			5.0	5.0	100.0			
	310.1799	310.1807	-0.8	-2.6	9.5	821.4	0.0	C20 H24 N O2

12 ^{1}H NMR $CDCl_3$ 500MHz

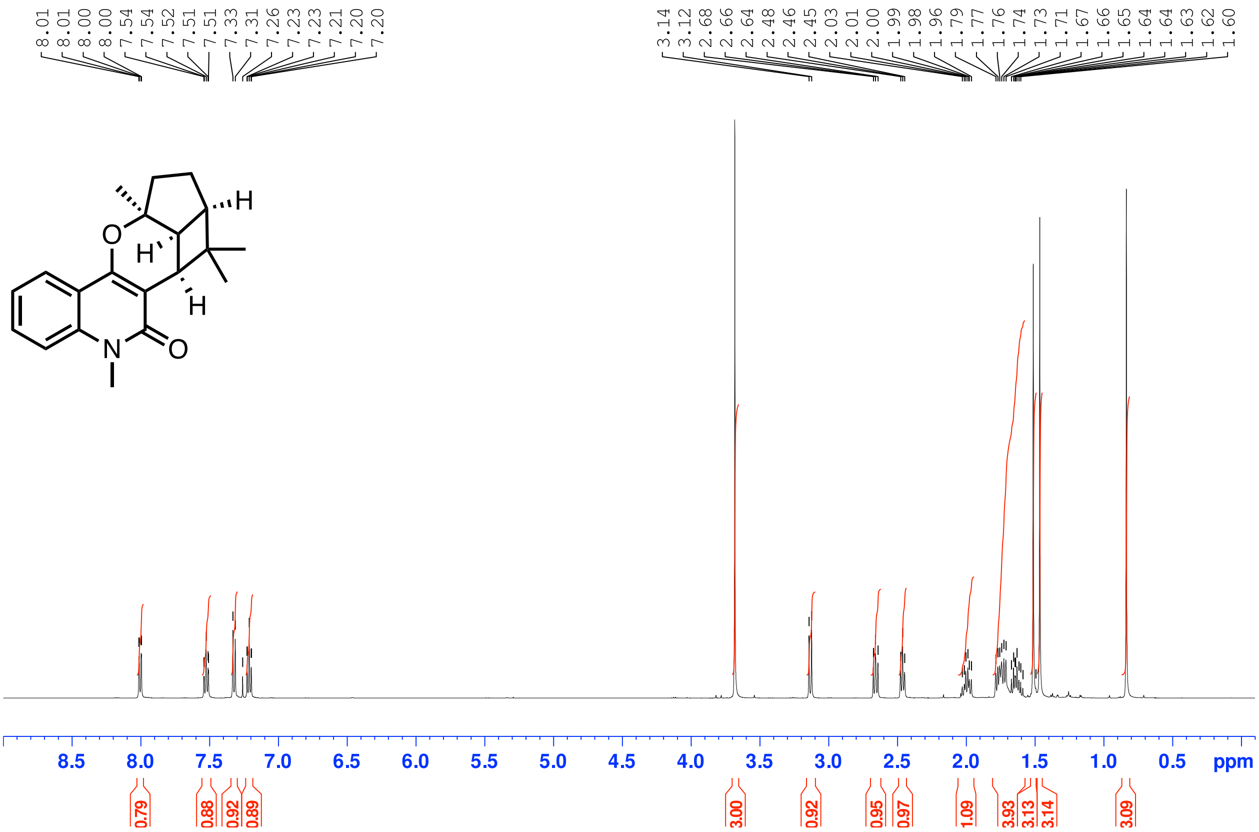

12 ^{13}C NMR $CDCl_3$ 125MHz

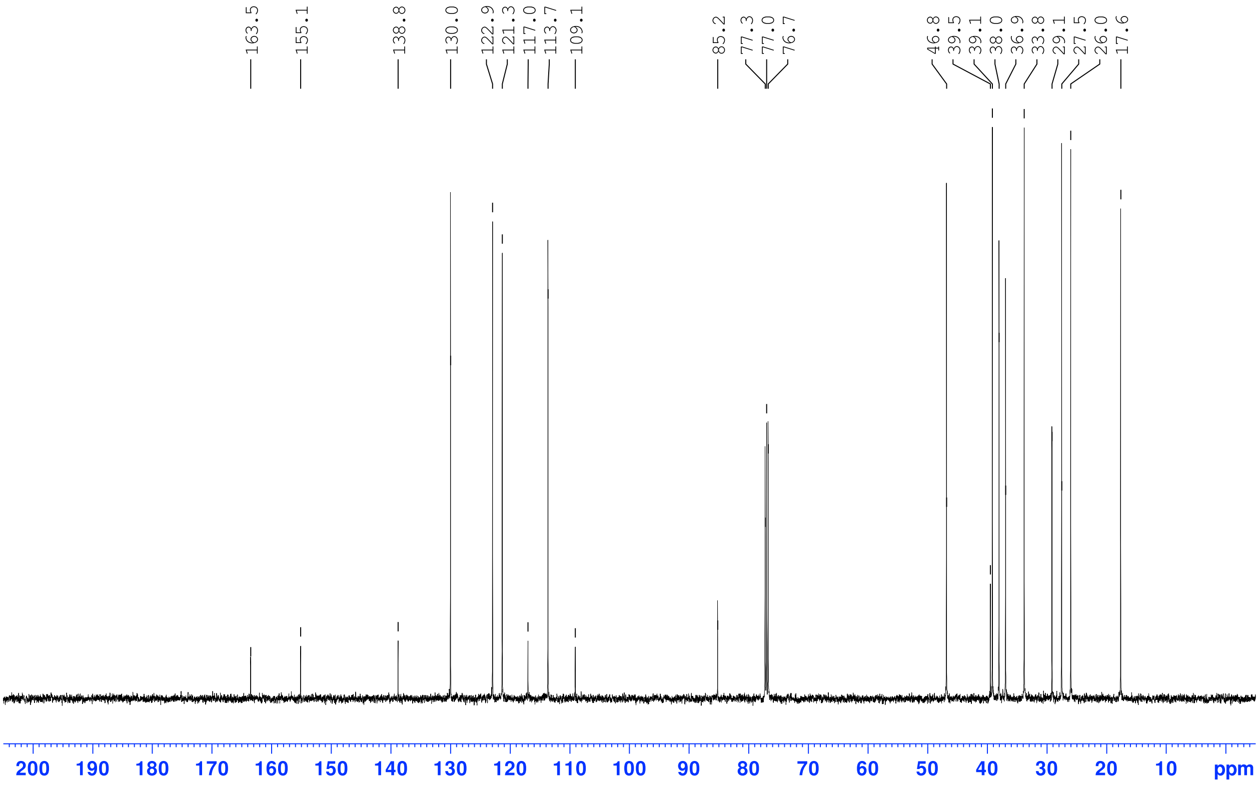

12 HRMS (ESI+)

Single Mass Analysis
Tolerance = 5.0 PPM / DBE: min = -1.5, max = 100.0
Element prediction: Off
Number of isotope peaks used for i-FIT = 9

Monoisotopic Mass, Even Electron Ions
265 formula(e) evaluated with 1 results within limits (all results (up to 1000) for each mass)
Elements Used:
C: 0-100 H: 0-120 N: 0-6 O: 0-10

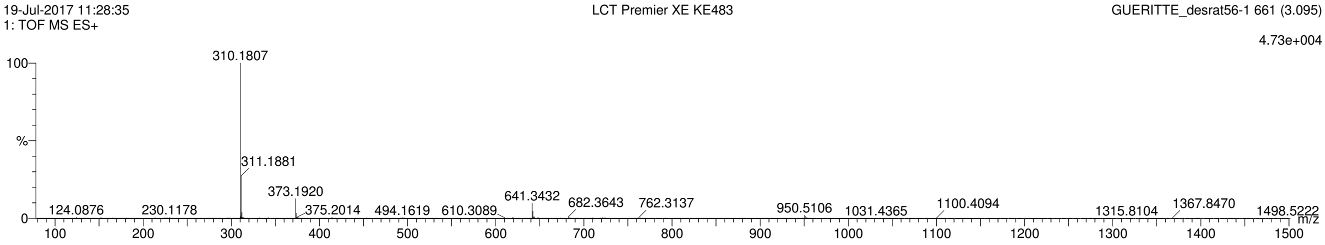

Minimum:				-1.5			
Maximum:		5.0	5.0	100.0			
Mass	Calc. Mass	mDa	PPM	DBE	i-FIT	i-FIT (Norm)	Formula
310.1807	310.1807	0.0	0.0	9.5	692.1	0.0	C20 H24 N O2

14 ^{1}H NMR $CDCl_3$ 500MHz

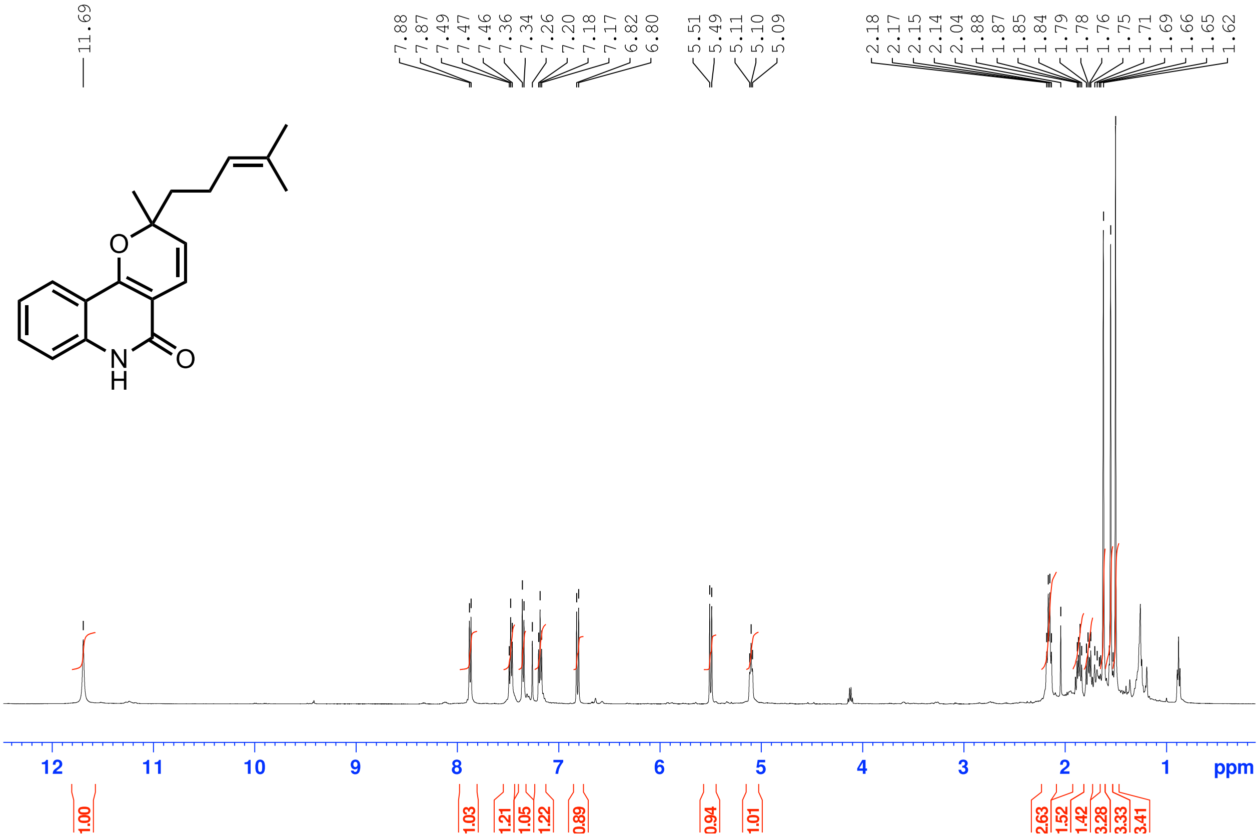

14 HRMS (ESI+)

Single Mass Analysis
Tolerance = 5.0 PPM / DBE: min = -1.5, max = 100.0
Element prediction: Off
Number of isotope peaks used for i-FIT = 9

Monoisotopic Mass, Even Electron Ions
115 formula(e) evaluated with 1 results within limits (all results (up to 1000) for each mass)
Elements Used:
C: 0-100 H: 0-120 N: 0-2 O: 0-10

02-Mar-2018 14:30:23 LCT Premier XE KE483 GUERITTE_desrat64-1 584 (2.728)
1: TOF MS ES+ 5.05e+004

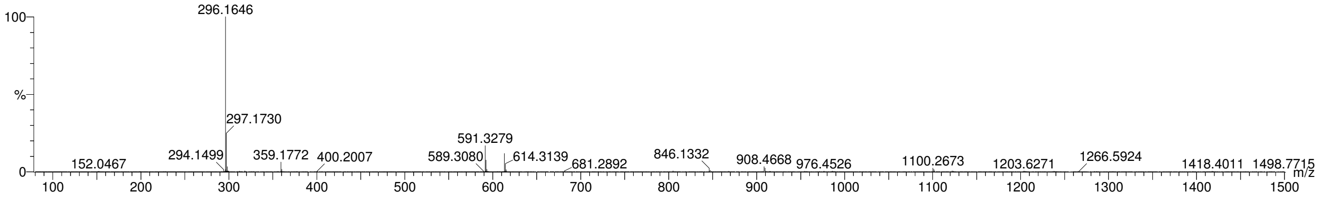

Minimum:				-1.5			
Maximum:		5.0	5.0	100.0			
Mass	Calc. Mass	mDa	PPM	DBE	i-FIT	i-FIT (Norm)	Formula
296.1646	296.1651	-0.5	-1.7	9.5	761.7	0.0	C19 H22 N O2

15a ^{1}H NMR $CDCl_3$ 500MHz

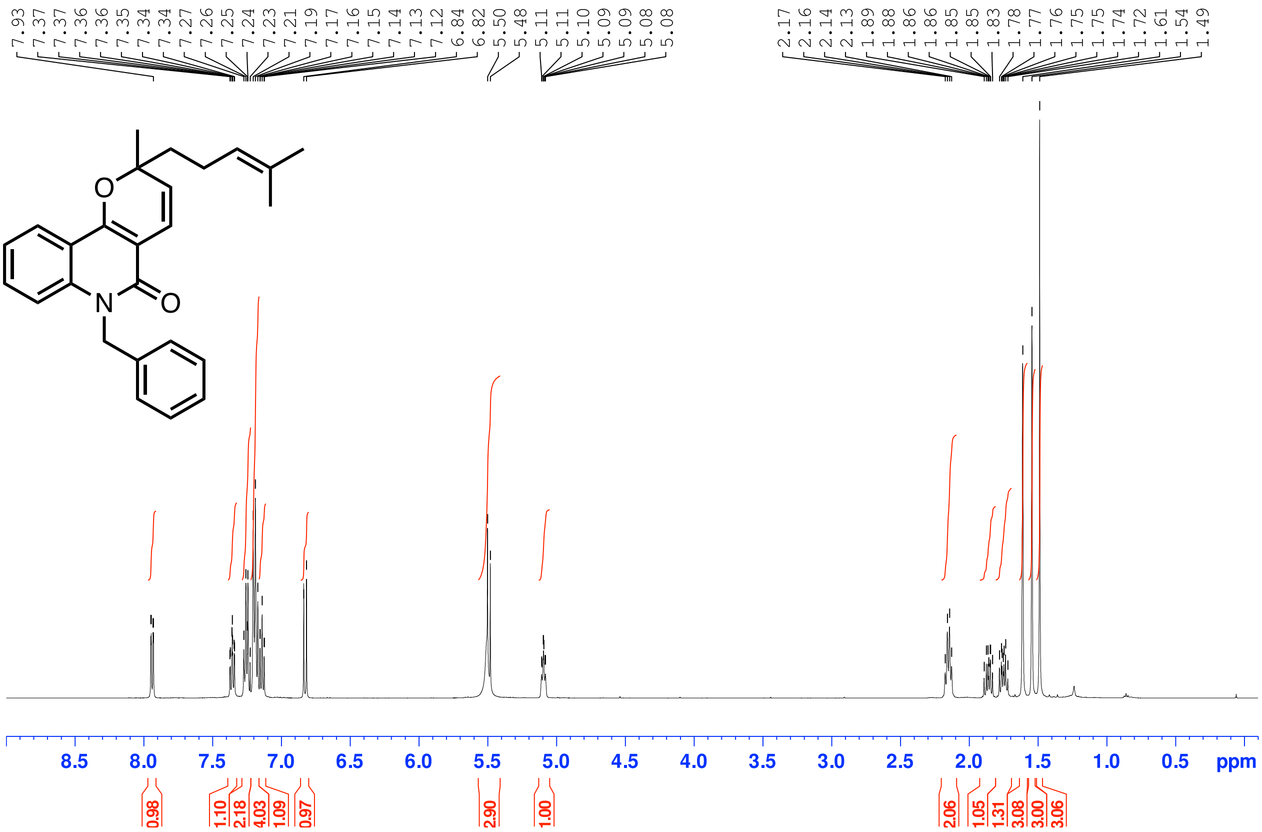

15a ^{13}C NMR $CDCl_3$ 125MHz

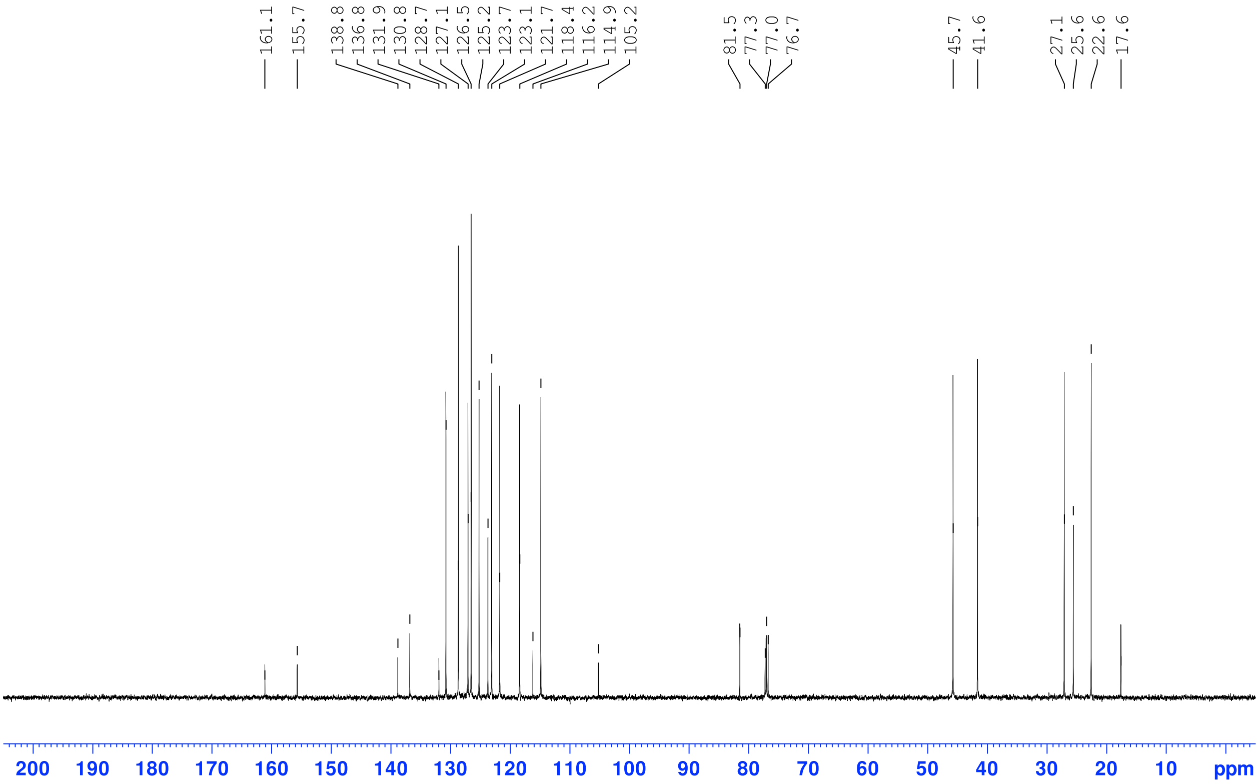

15a HRMS (ESI+)

Single Mass Analysis
Tolerance = 5.0 PPM / DBE: min = -1.5, max = 100.0
Element prediction: Off
Number of isotope peaks used for i-FIT = 9

Monoisotopic Mass, Even Electron Ions
652 formula(e) evaluated with 3 results within limits (all results (up to 1000) for each mass)
Elements Used:
C: 1-100 H: 0-100 N: 0-10 O: 0-16

19-Feb-2021 17:45:39 LCT Premier ROUSSI_apel7-2 20 (0.534)
1: TOF MS ES+

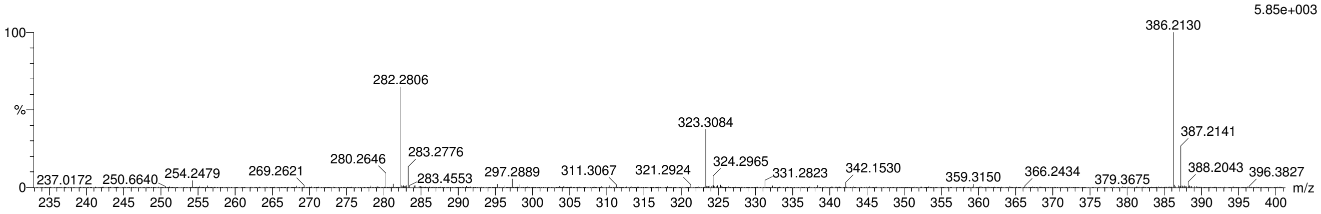

Minimum:				-1.5			
Maximum:		5.0	5.0	100.0			
Mass	Calc. Mass	mDa	PPM	DBE	i-FIT	i-FIT (Norm)	Formula
386.2130	386.2139	-0.9	-2.3	0.5	275.3	1.7	C14 H32 N3 O9
	386.2120	1.0	2.6	13.5	275.6	2.0	C26 H28 N O2
	386.2112	1.8	4.7	1.5	274.0	0.4	C10 H28 N9 O7

15b ^{1}H NMR $CDCl_3$ 500MHz

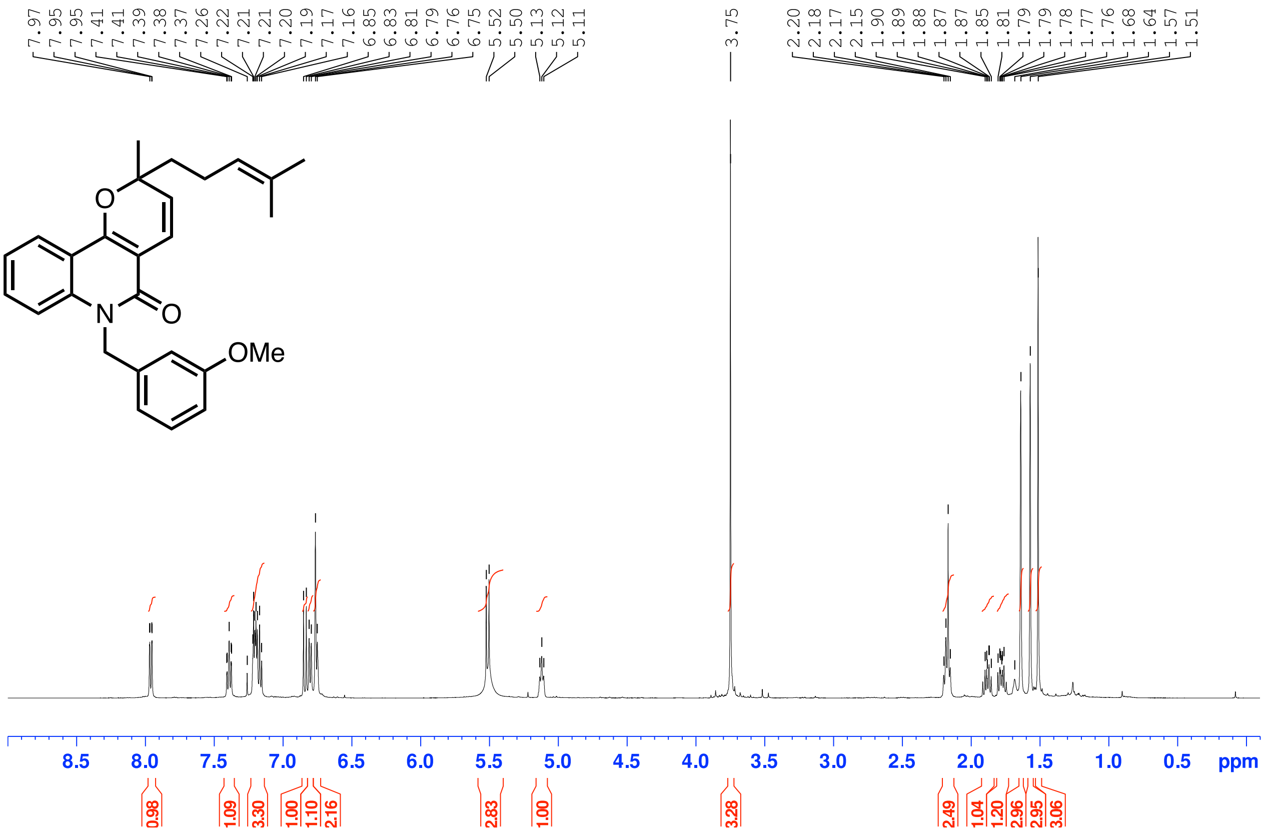

15b ^{13}C NMR $CDCl_3$ 125MHz

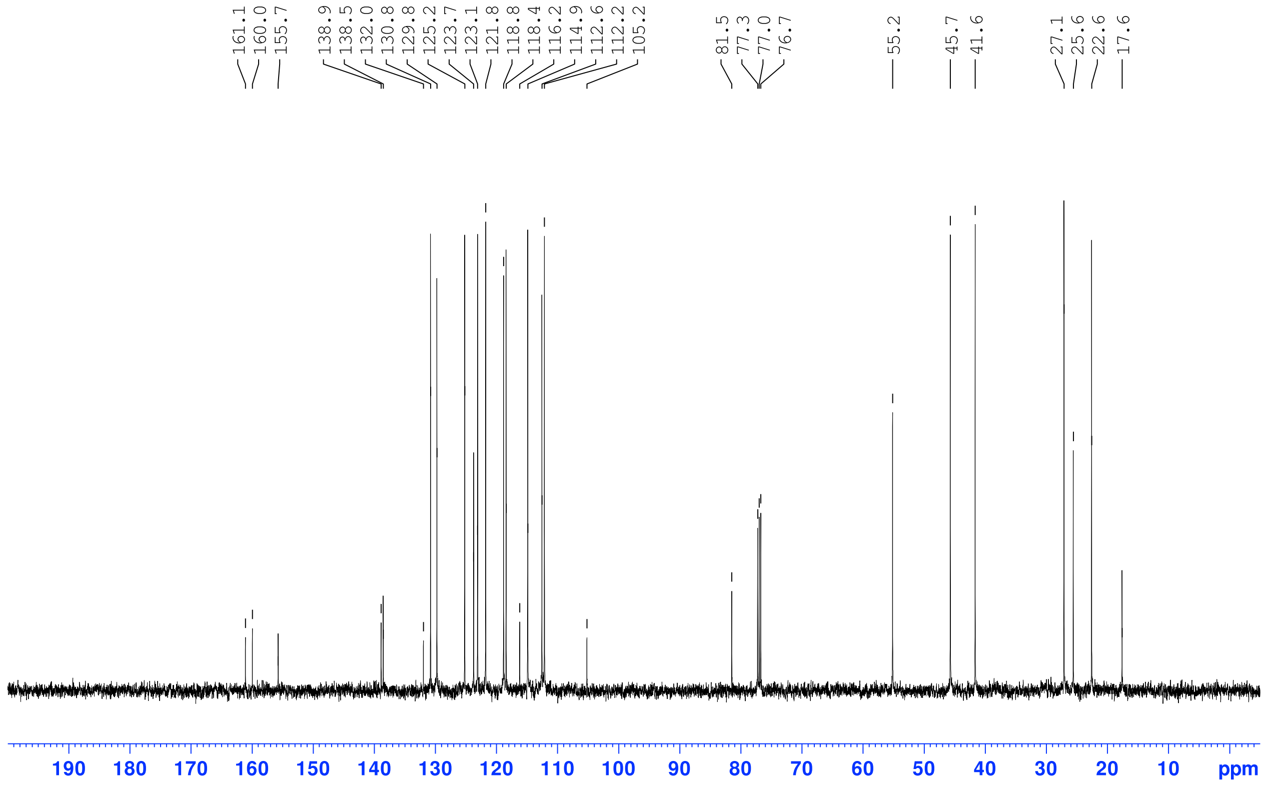

15b HRMS (ESI+)

Single Mass Analysis
Tolerance = 5.0 PPM / DBE: min = -1.5, max = 100.0
Element prediction: Off
Number of isotope peaks used for i-FIT = 9

Monoisotopic Mass, Even Electron Ions
422 formula(e) evaluated with 2 results within limits (all results (up to 1000) for each mass)
Elements Used:
C: 1-100 H: 0-100 N: 0-5 O: 0-16

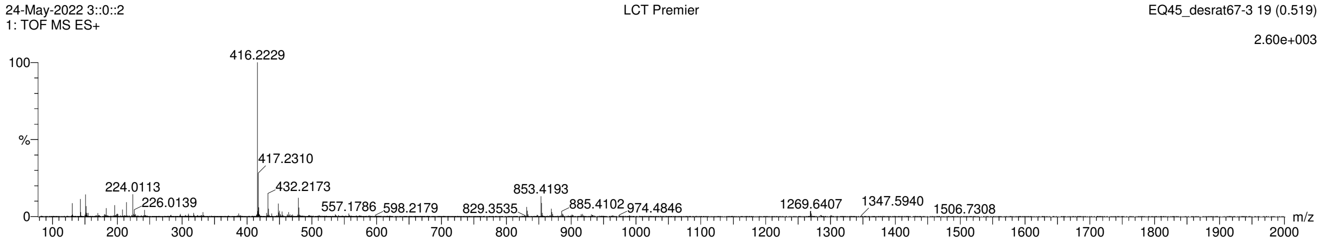

Minimum:				-1.5			
Maximum:		5.0	5.0	100.0			
Mass	Calc. Mass	mDa	PPM	DBE	i-FIT	i-FIT (Norm)	Formula
416.2229	416.2226	0.3	0.7	13.5	233.7	0.0	C27 H30 N O3
	416.2244	-1.5	-3.6	0.5	240.2	6.5	C15 H34 N3 O10

15c ^{1}H NMR $CDCl_3$ 500MHz

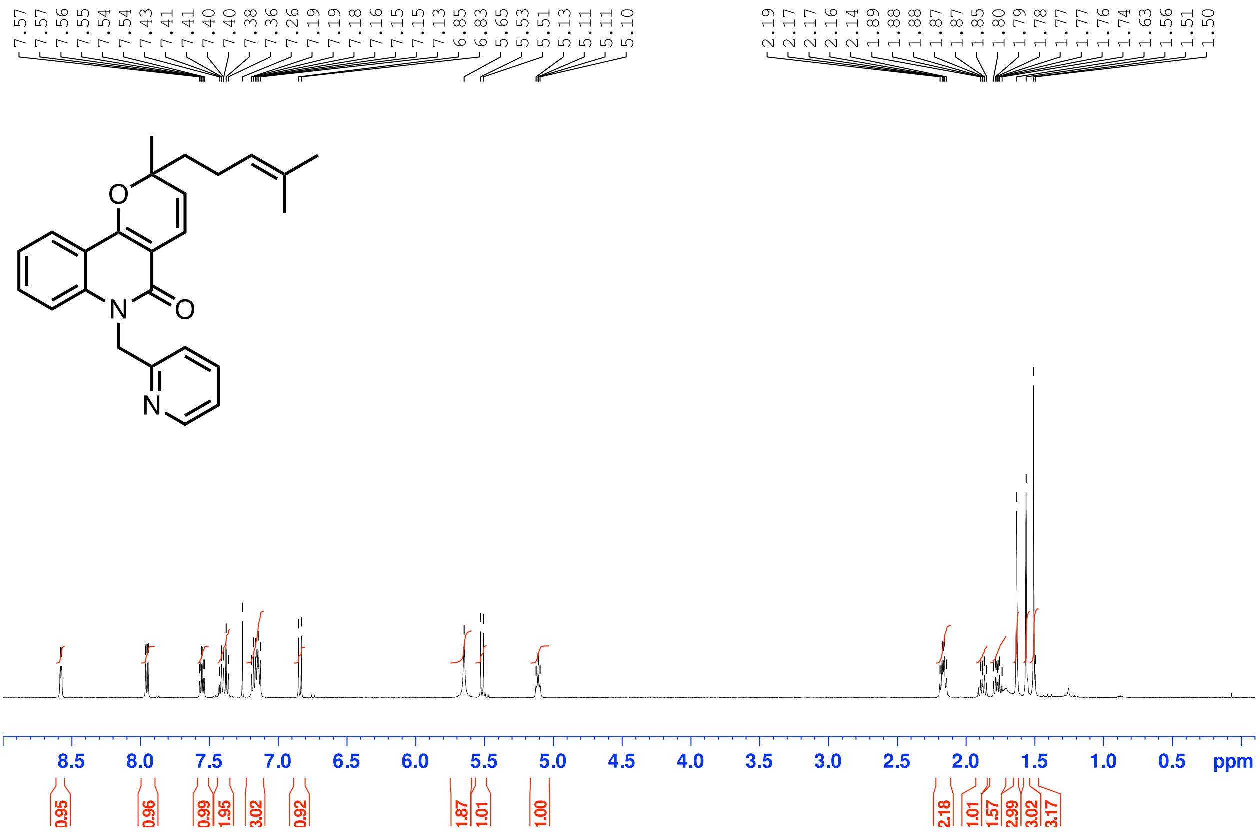

15c ^{13}C NMR $CDCl_3$ 125MHz

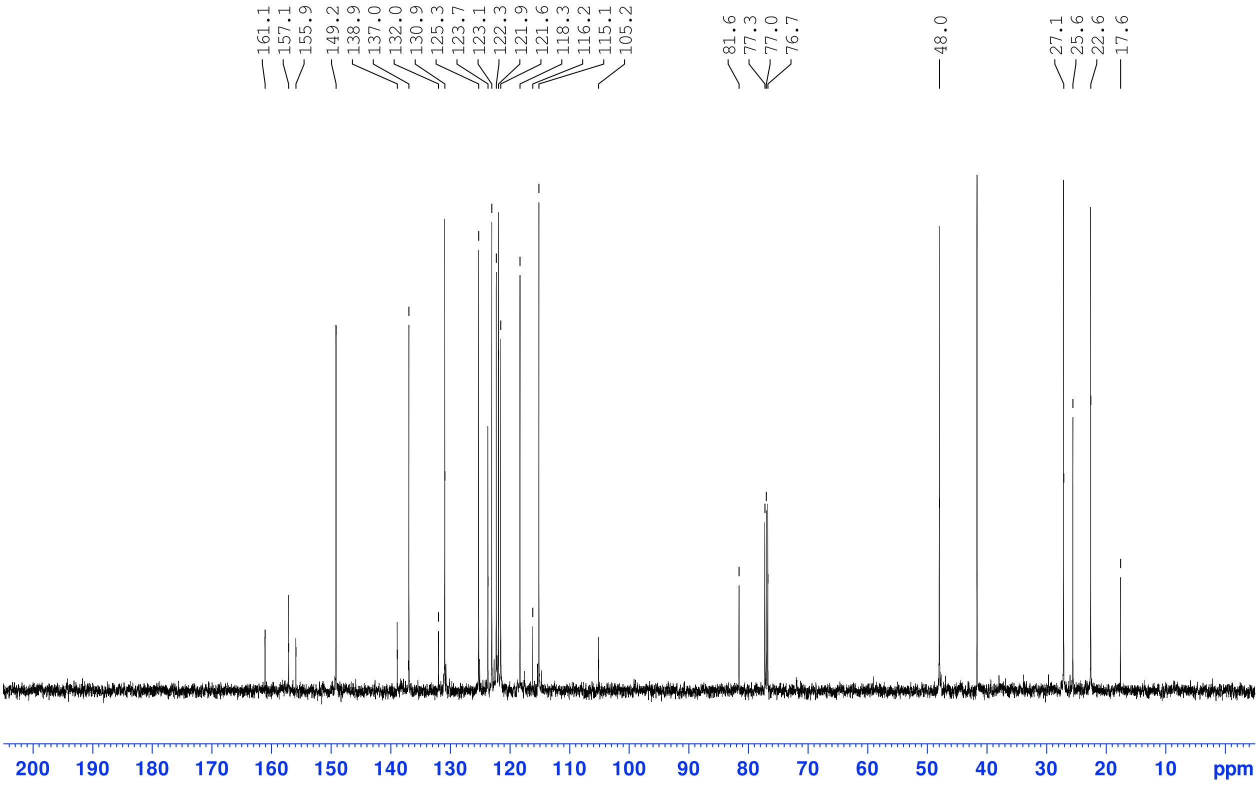

15c HRMS (ESI+)

Single Mass Analysis
Tolerance = 5.0 PPM / DBE: min = -1.5, max = 100.0
Element prediction: Off
Number of isotope peaks used for i-FIT = 9

Monoisotopic Mass, Even Electron Ions
395 formula(e) evaluated with 1 results within limits (all results (up to 1000) for each mass)
Elements Used:
C: 1-100 H: 0-100 N: 0-5 O: 0-16

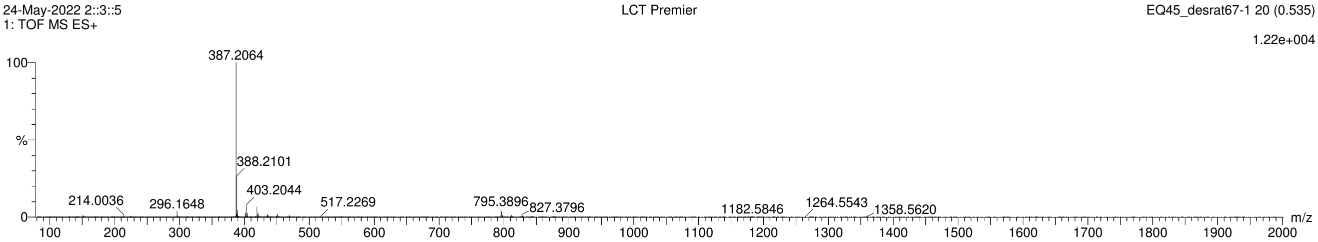

Minimum:				-1.5			
Maximum:		5.0	5.0	100.0			

Mass	Calc. Mass	mDa	PPM	DBE	i-FIT	i-FIT (Norm)	Formula
387.2064	387.2073	-0.9	-2.3	13.5	545.4	0.0	C25 H27 N2 O2

15d ^{1}H NMR $CDCl_3$ 500MHz

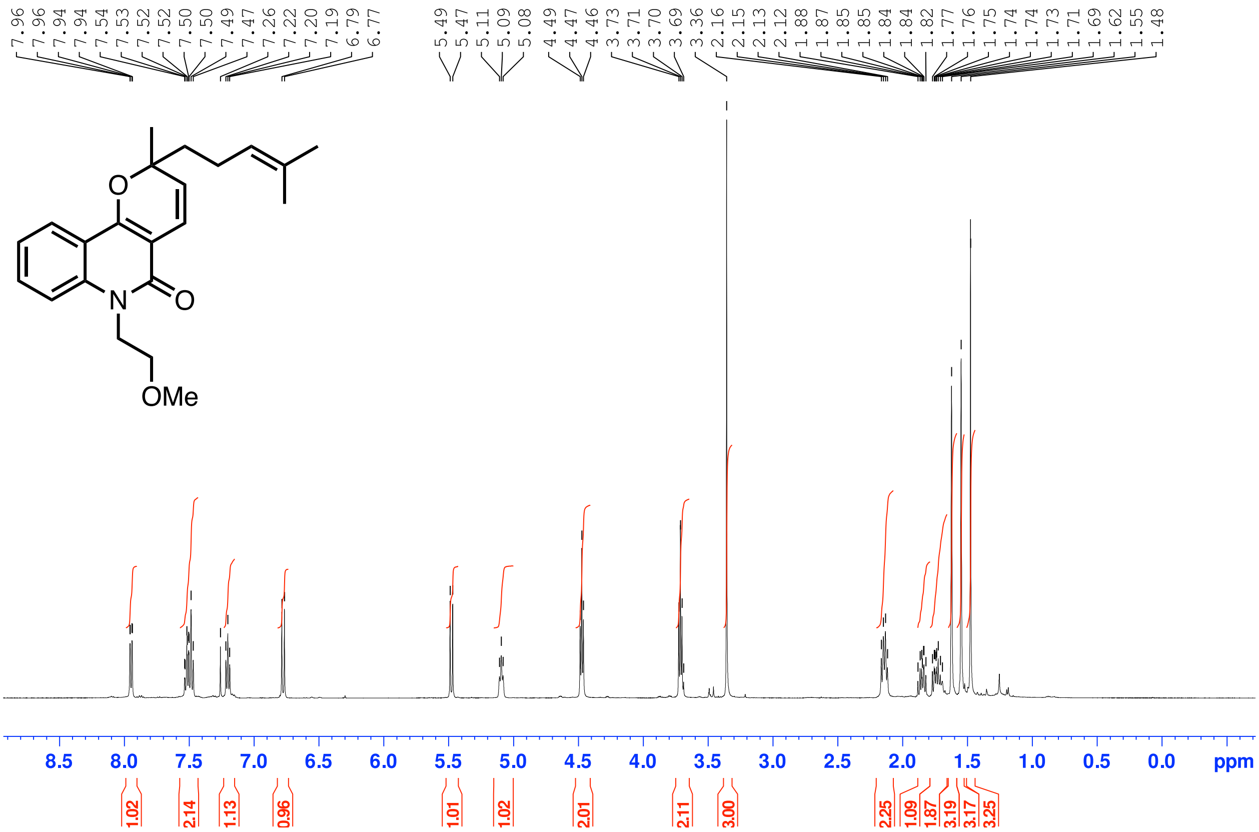

15d ^{13}C NMR $CDCl_3$ 125MHz

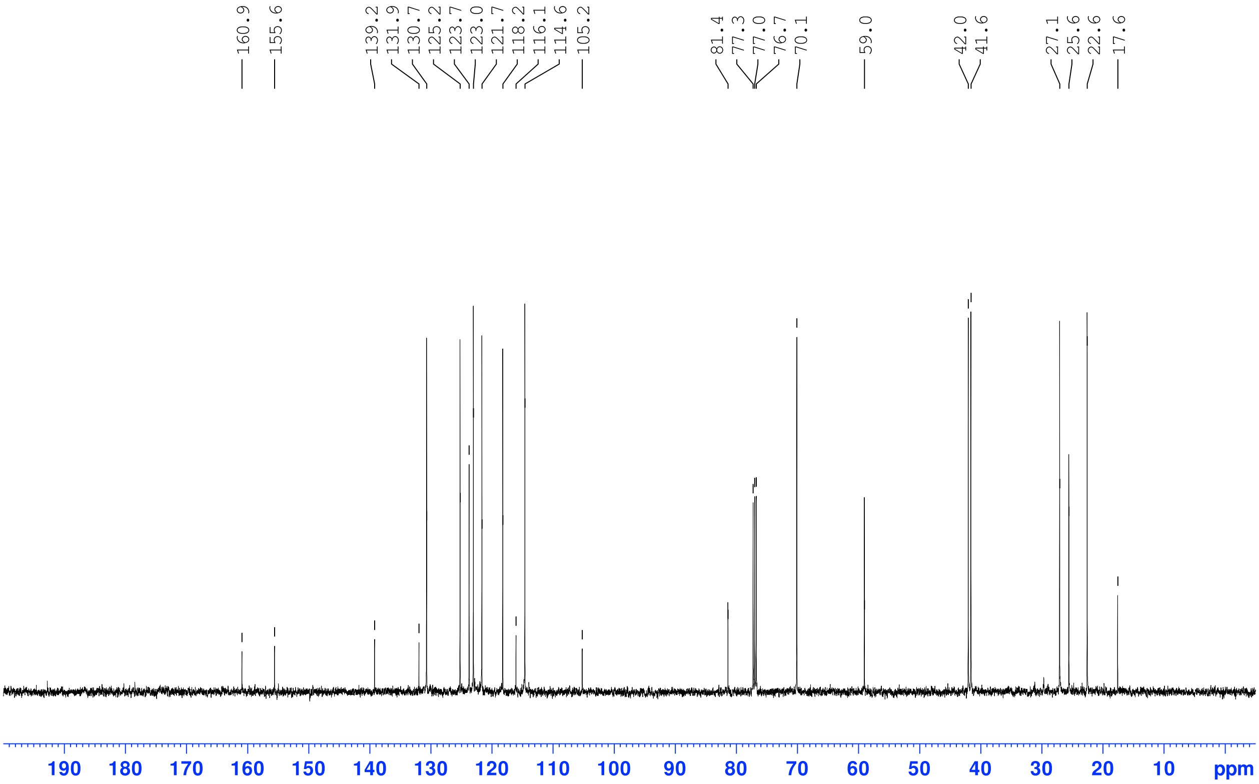

15d HRMS (ESI+)

Single Mass Analysis
Tolerance = 20.0 mDa / DBE: min = -1.5, max = 100.0
Element prediction: Off
Number of isotope peaks used for i-FIT = 9

Monoisotopic Mass, Even Electron Ions
58 formula(e) evaluated with 0 results within limits (all results (up to 1000) for each mass)
Elements Used:
C: 0-50 H: 0-120 O: 0-15

19-Feb-2021 17:51:40 LCT Premier ROUSSI_apel7-4 29 (0.751)
1: TOF MS ES+

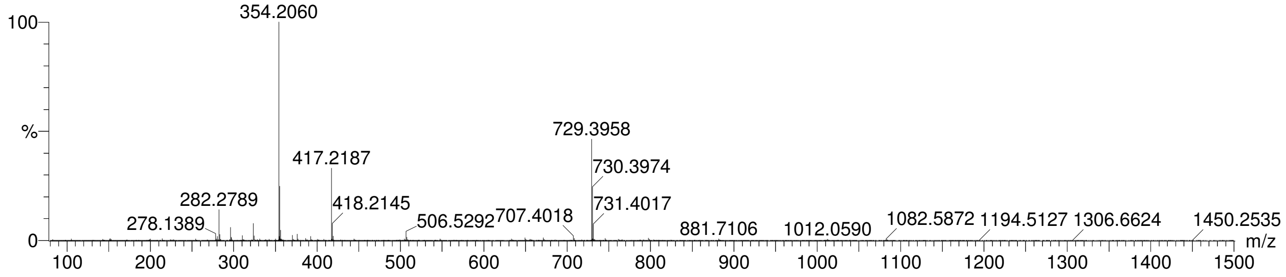

Minimum:				-1.5			
Maximum:		20.0	20.0	100.0			
Mass	Calc. Mass	mDa	PPM	DBE	i-FIT	i-FIT (Norm)	Formula
354.2060	---						

15e ^{1}H NMR $CDCl_3$ 500MHz

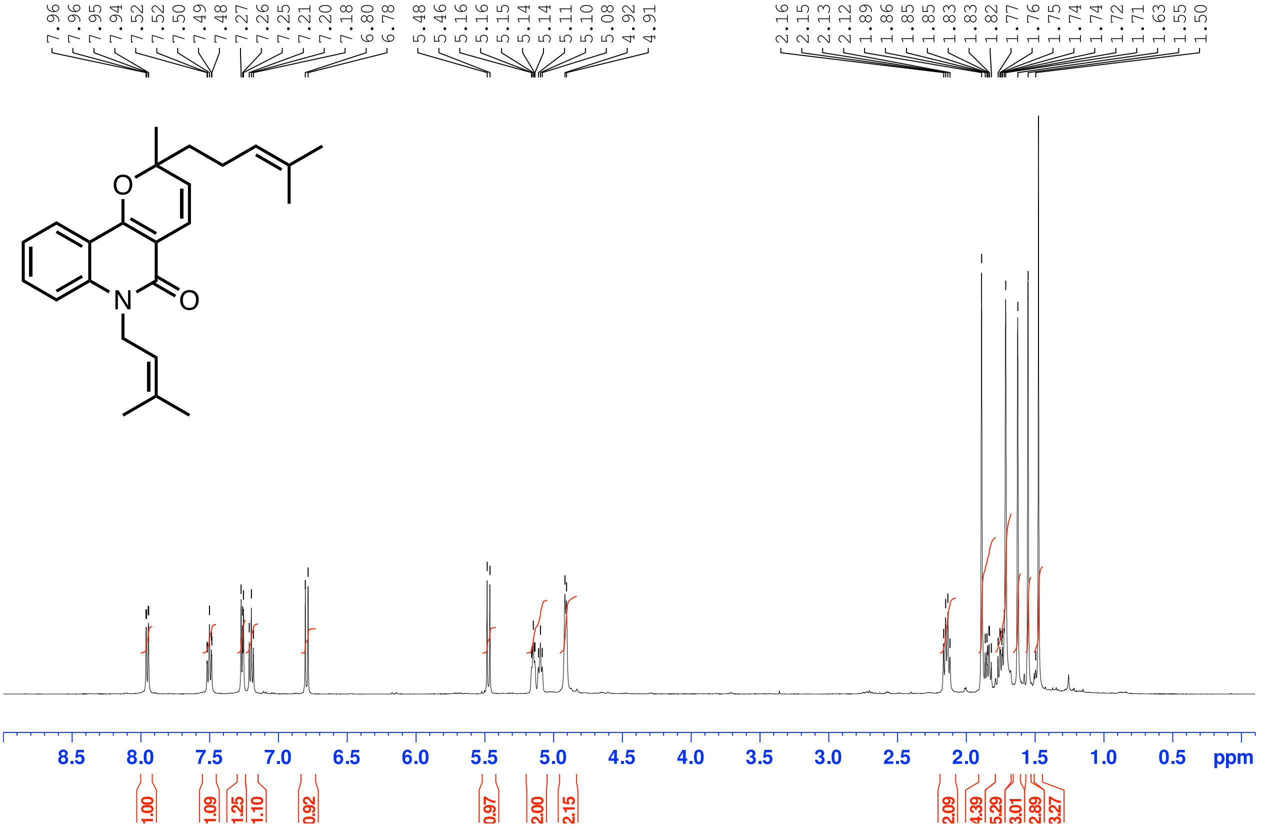

15e ^{13}C NMR $CDCl_3$ 125MHz

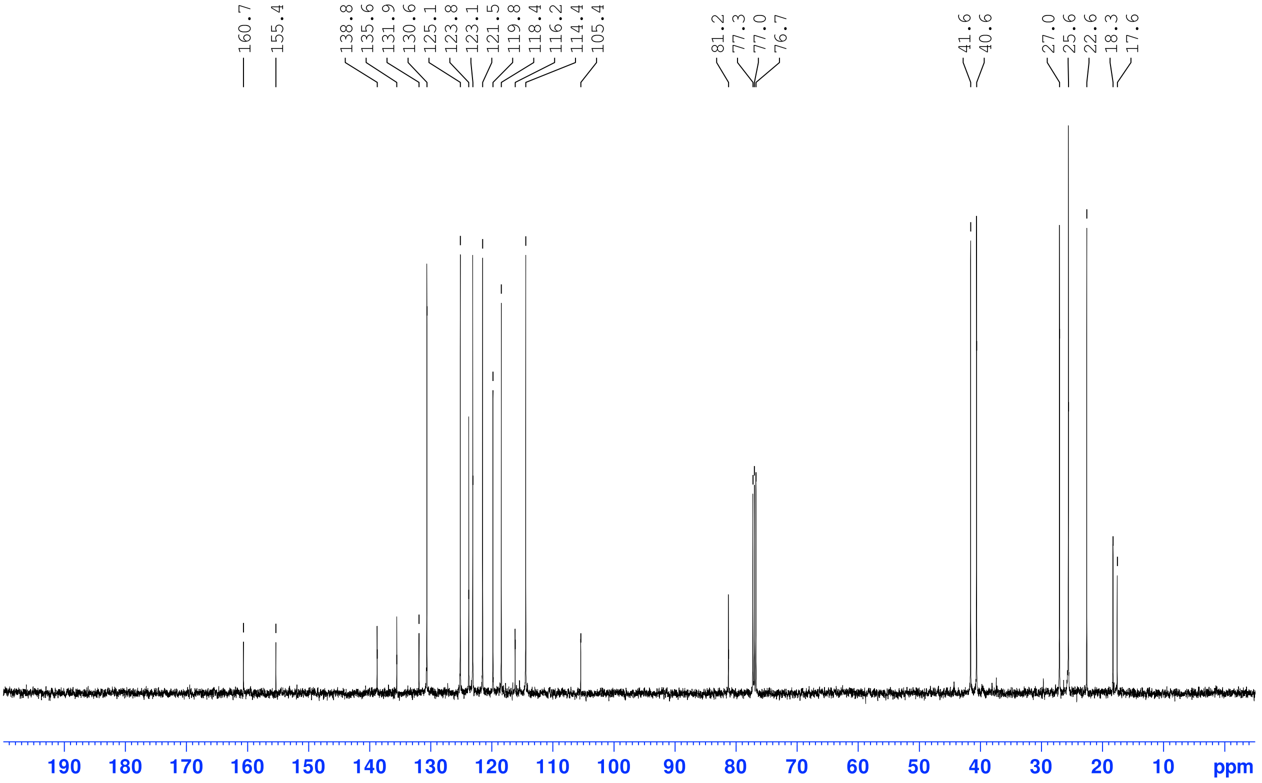

15e HRMS (ESI+)

Single Mass Analysis

Tolerance = 20.0 mDa / DBE: min = -1.5, max = 100.0
Element prediction: Off
Number of isotope peaks used for i-FIT = 9

Monoisotopic Mass, Even Electron Ions
63 formula(e) evaluated with 0 results within limits (all results (up to 1000) for each mass)
Elements Used:
C: 0-50 H: 0-120 O: 0-15

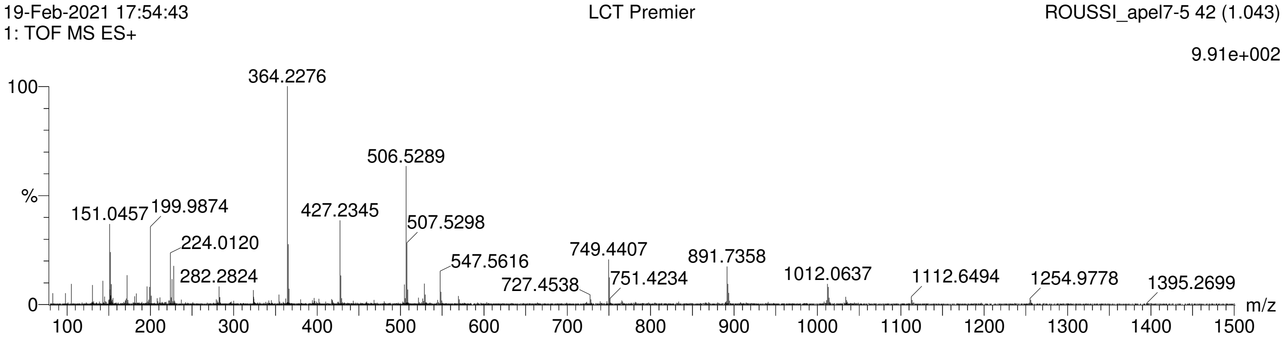

Minimum:				-1.5			
Maximum:		20.0	20.0	100.0			
Mass	Calc. Mass	mDa	PPM	DBE	i-FIT	i-FIT (Norm)	Formula
364.2276	---						

15f ^{1}H NMR $CDCl_3$ 500MHz

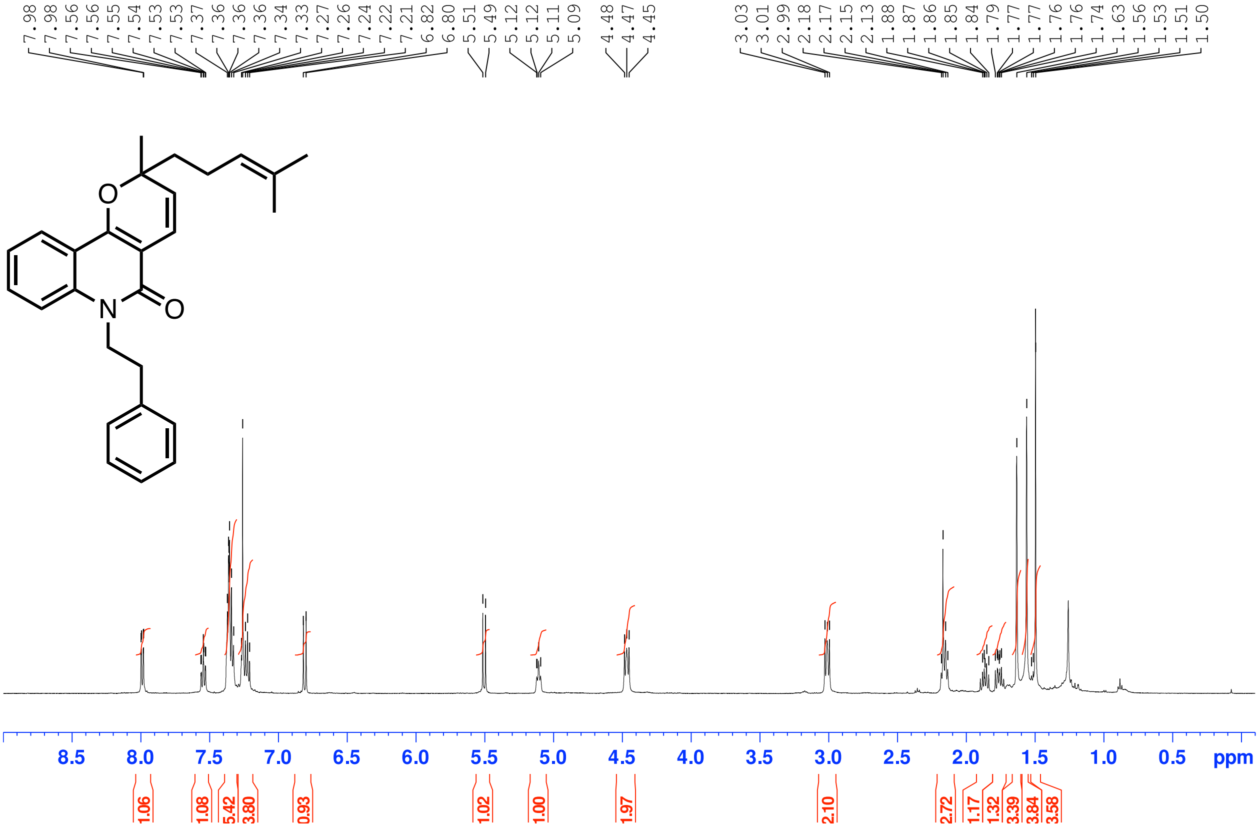

15f ^{13}C NMR $CDCl_3$ 125MHz

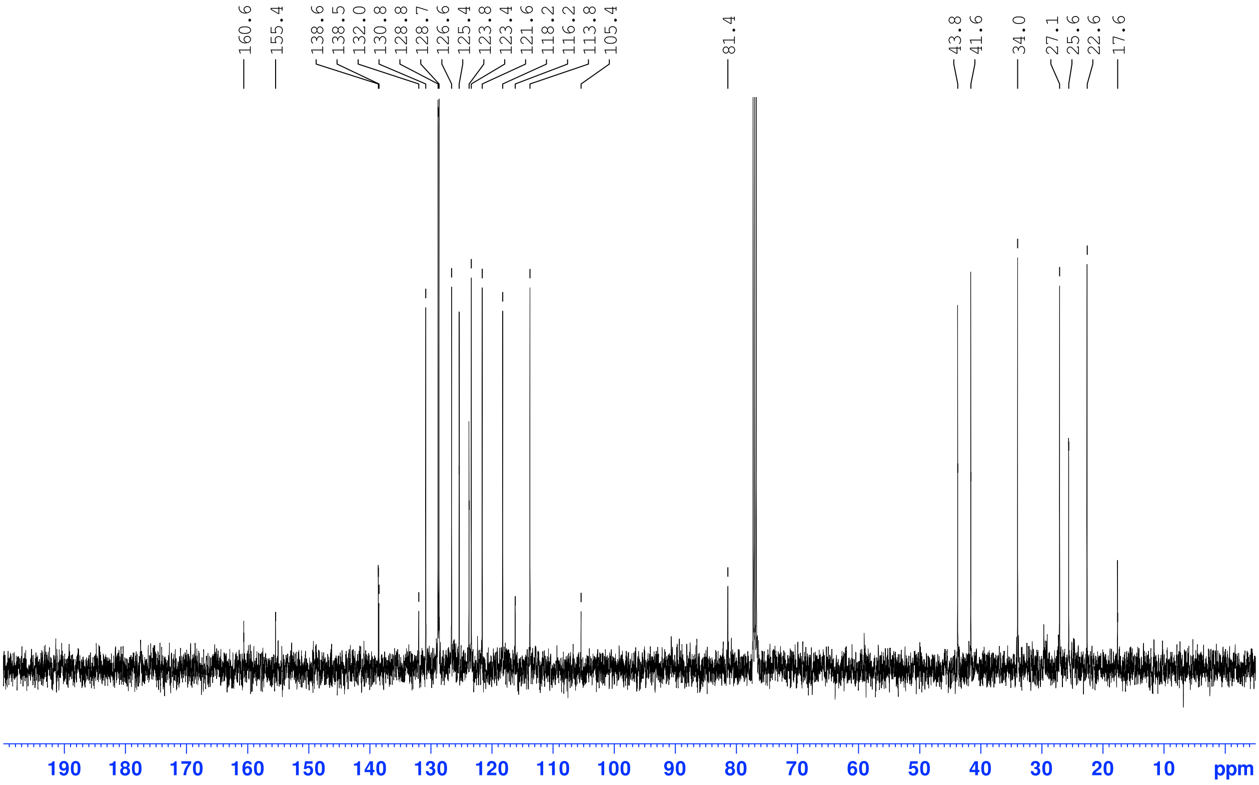

15f HRMS (ESI+)

Single Mass Analysis
Tolerance = 20.0 mDa / DBE: min = -1.5, max = 100.0
Element prediction: Off
Number of isotope peaks used for i-FIT = 9

Monoisotopic Mass, Even Electron Ions
70 formula(e) evaluated with 0 results within limits (all results (up to 1000) for each mass)
Elements Used:
C: 0-50 H: 0-120 O: 0-15

19-Feb-2021 16:56:59 LCT Premier ROUSSI_apel5-1 20 (0.534)
1: TOF MS ES+

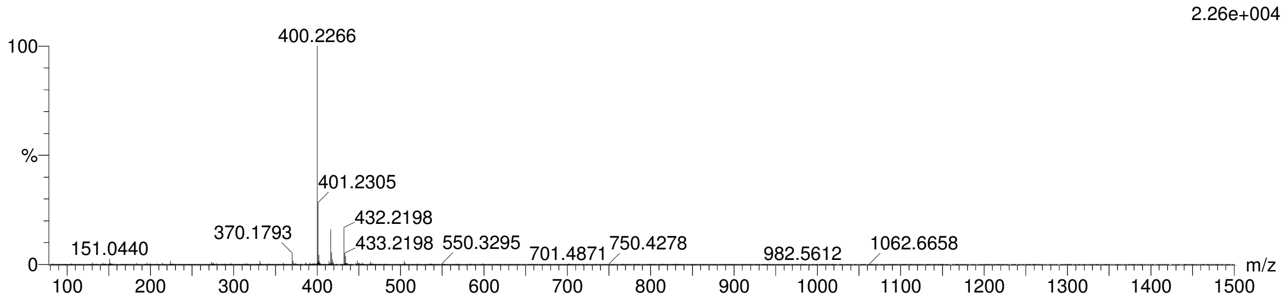

Minimum:				-1.5			
Maximum:		20.0	20.0	100.0			
Mass	Calc. Mass	mDa	PPM	DBE	i-FIT	i-FIT (Norm)	Formula
400.2266	---						

15g ¹H NMR $CDCl_3$ 500MHz

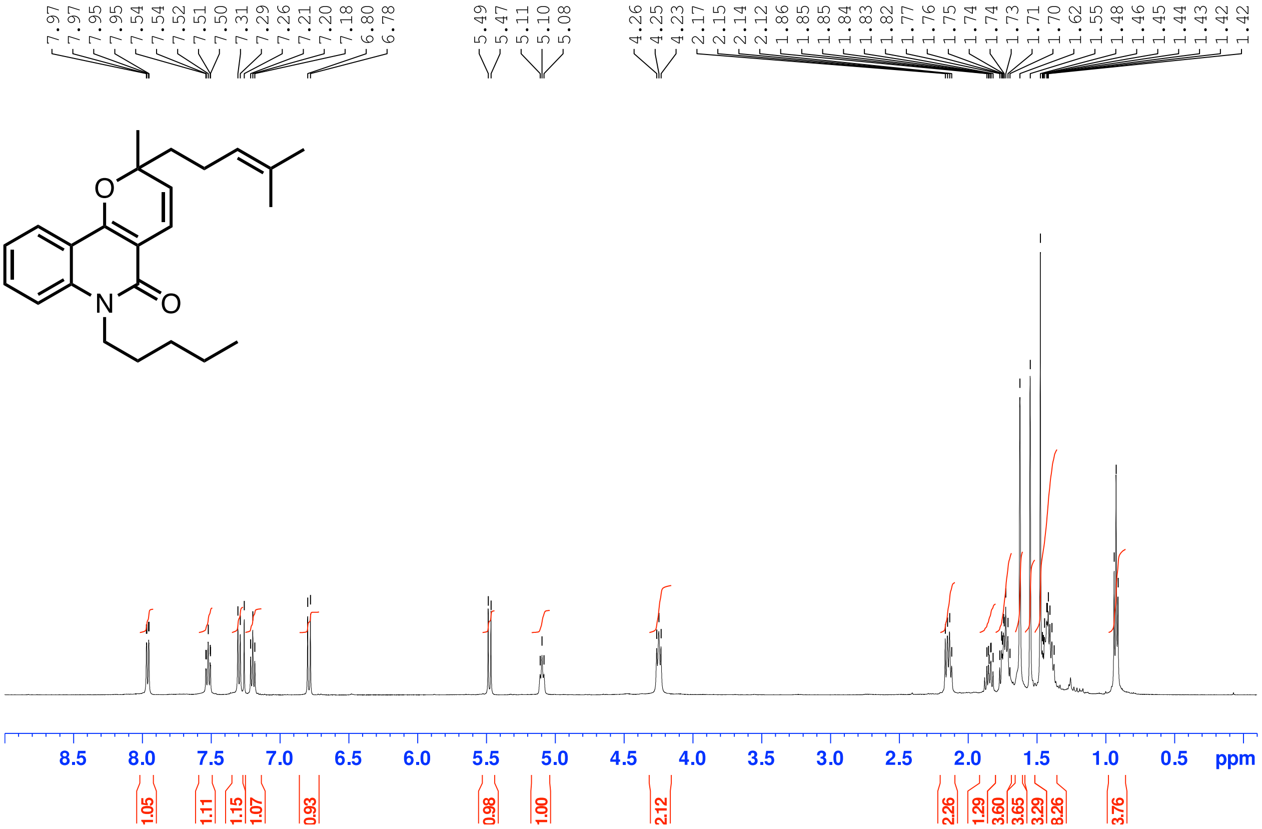

15g ¹³C NMR $CDCl_3$ 125MHz

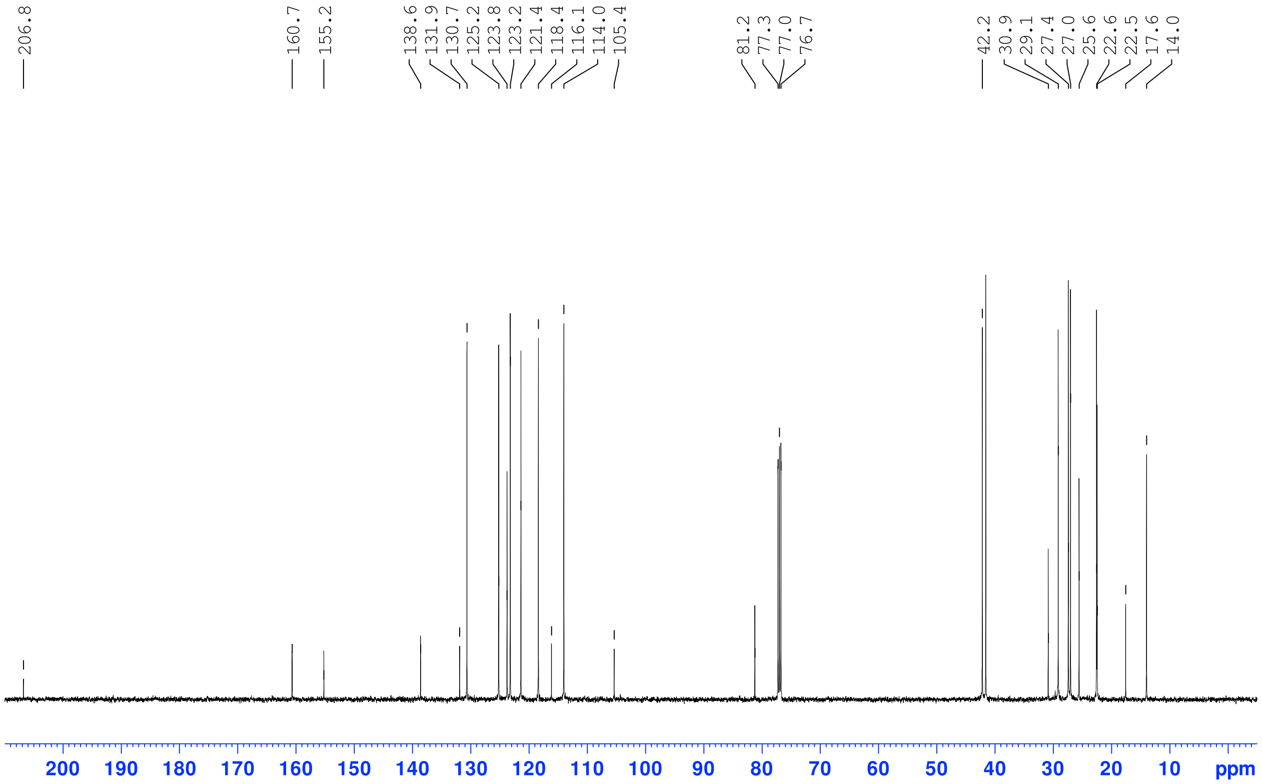

15g HRMS (ESI+)

Single Mass Analysis
Tolerance = 5.0 PPM / DBE: min = -1.5, max = 100.0
Element prediction: Off
Number of isotope peaks used for i-FIT = 9

Monoisotopic Mass, Even Electron Ions
608 formula(e) evaluated with 1 results within limits (all results (up to 1000) for each mass)
Elements Used:
C: 1-100 H: 0-100 N: 0-10 O: 0-16

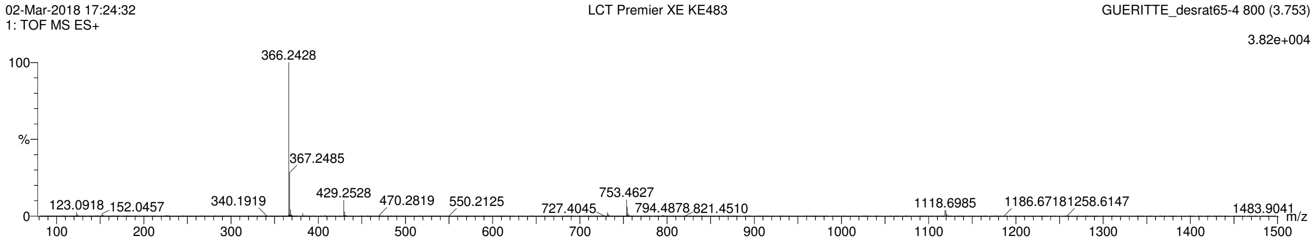

Minimum:				-1.5			
Maximum:		5.0	5.0	100.0			
Mass	Calc. Mass	mDa	PPM	DBE	i-FIT	i-FIT (Norm)	Formula
366.2428	366.2433	-0.5	-1.4	9.5	622.0	0.0	C24 H32 N O2

15h ^{1}H NMR $CDCl_3$ 500MHz

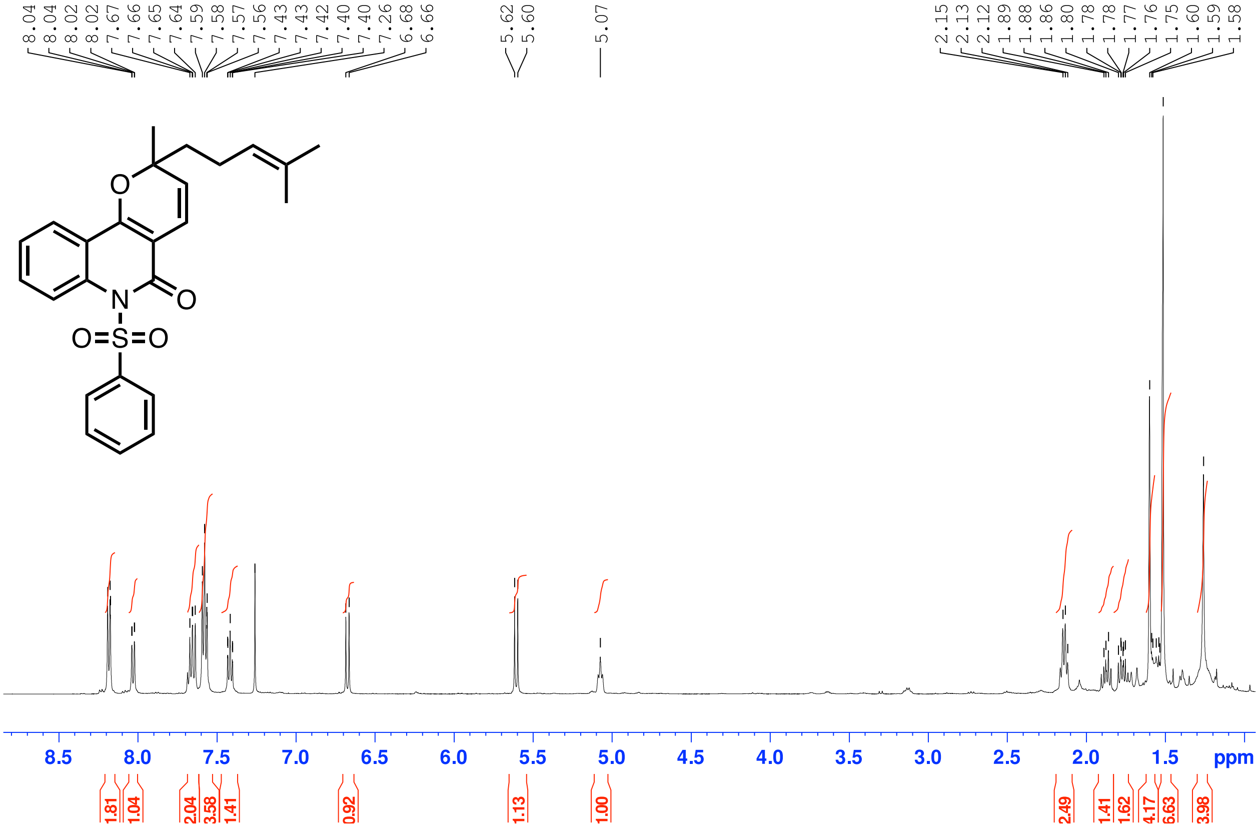

15h ^{13}C NMR $CDCl_3$ 125MHz

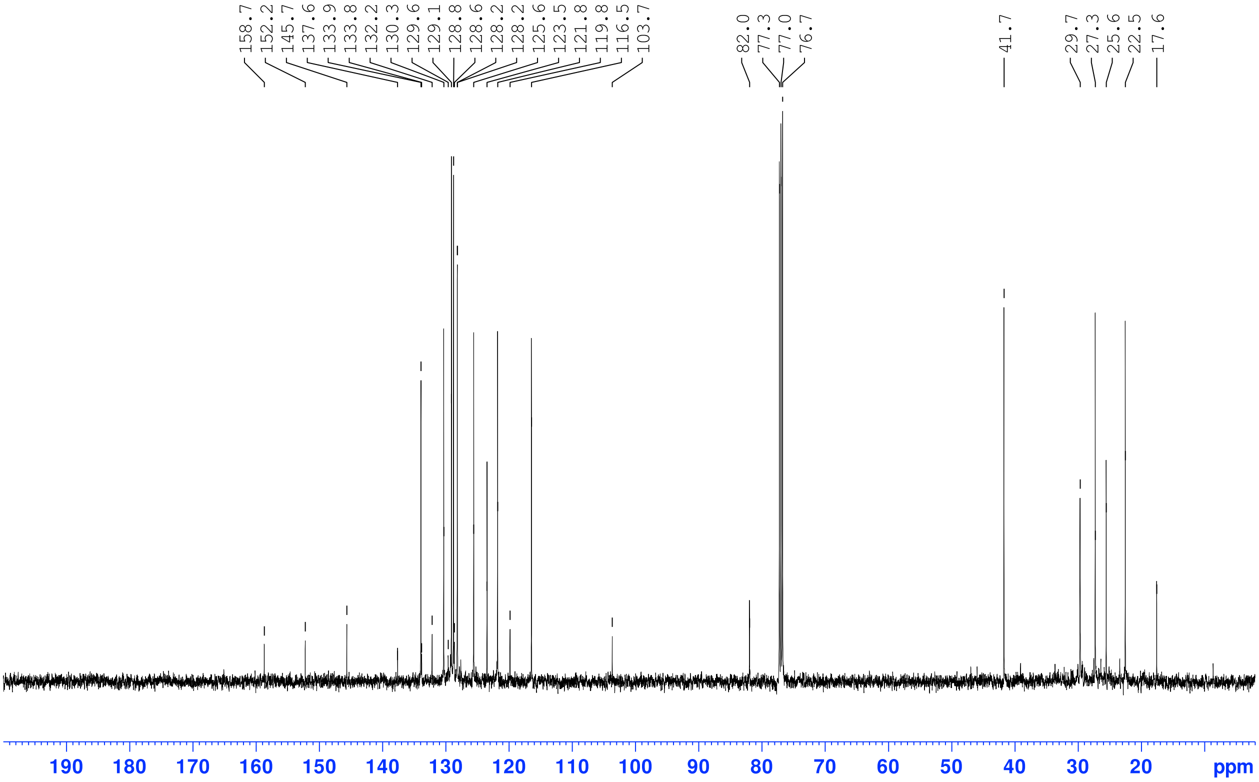

15h HRMS (ESI+)

Single Mass Analysis
Tolerance = 5.0 PPM / DBE: min = -1.5, max = 100.0
Element prediction: Off
Number of isotope peaks used for i-FIT = 9

Monoisotopic Mass, Even Electron Ions
706 formula(e) evaluated with 3 results within limits (all results (up to 1000) for each mass)
Elements Used:
C: 1-100 H: 0-100 N: 0-10 O: 0-16 S: 1-1

10-Apr-2018 4::5::5 LCT Premier XE KE483 ROUSSI_martin1-1 735 (3.438)
1: TOF MS ES+ 6.85e+003

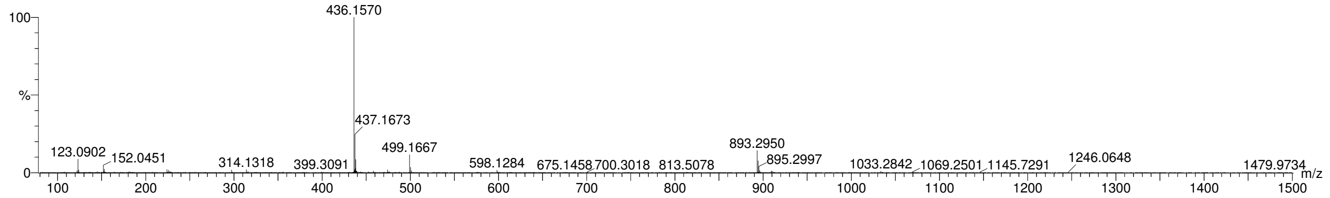

Minimum:				-1.5			
Maximum:		5.0	5.0	100.0			

Mass	Calc. Mass	mDa	PPM	DBE	i-FIT	i-FIT (Norm)	Formula
436.1570	436.1583	-1.3	-3.0	13.5	296.7	2.3	C25 H26 N O4 S
	436.1556	1.4	3.2	14.5	294.4	0.1	C21 H22 N7 O2 S
	436.1574	-0.4	-0.9	1.5	301.9	7.6	C9 H26 N9 O9 S

15i ^{1}H NMR $CDCl_3$ 500MHz

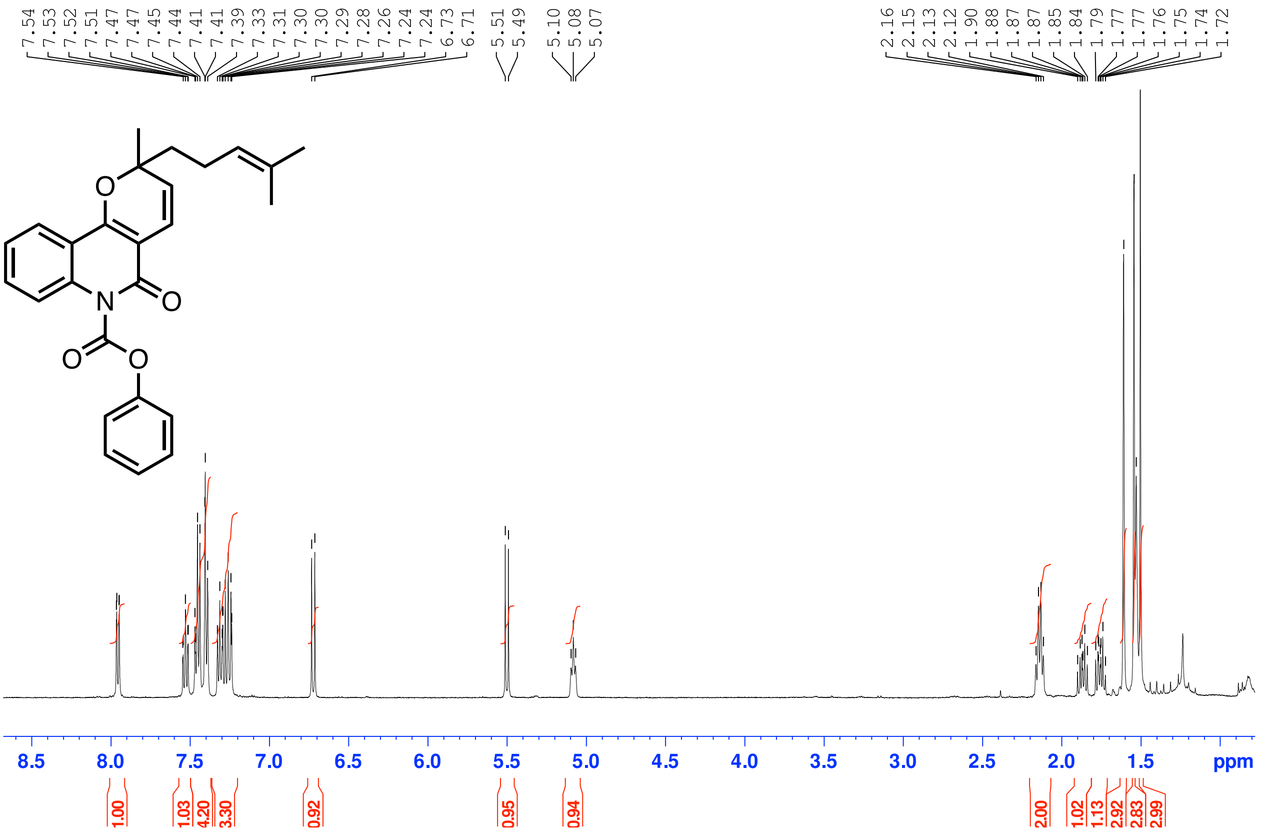

15i ^{13}C NMR $CDCl_3$ 125MHz

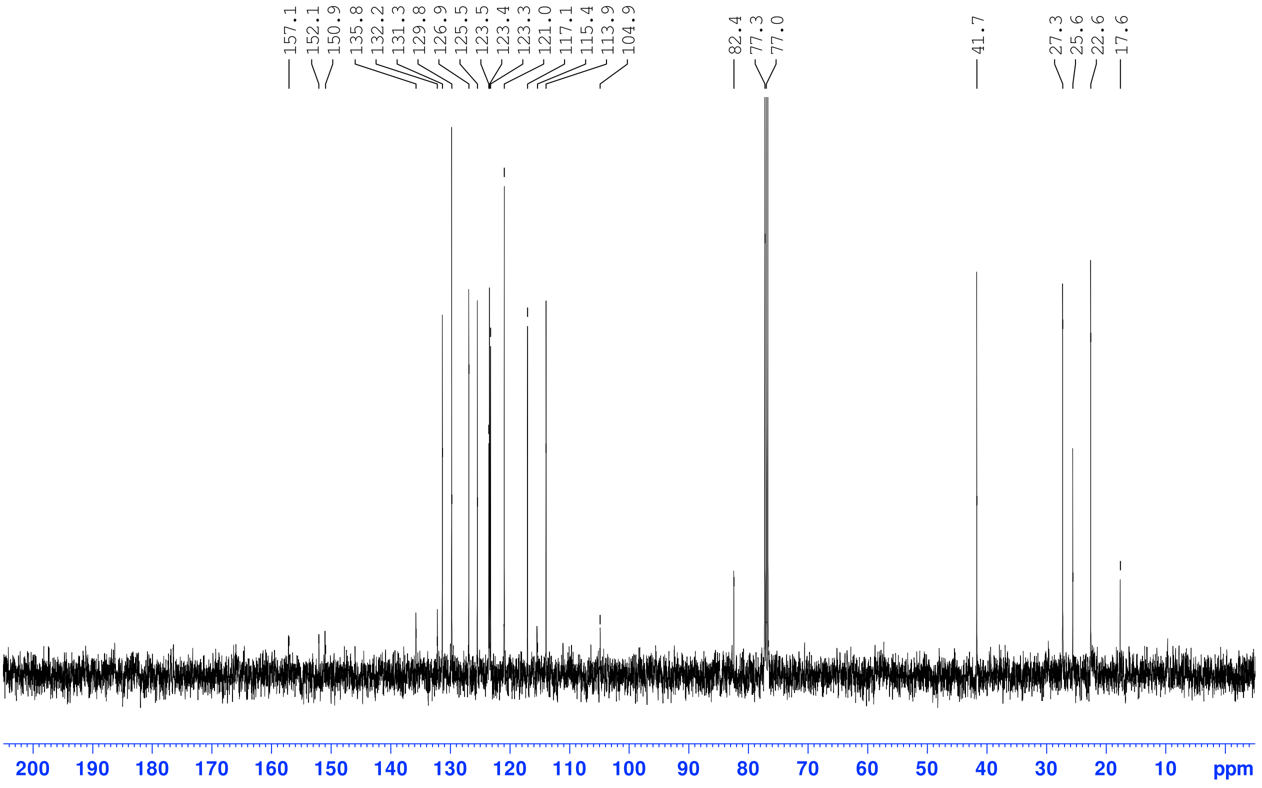

15i HRMS (ESI+)

Single Mass Analysis
Tolerance = 5.0 PPM / DBE: min = -1.5, max = 100.0
Element prediction: Off
Number of isotope peaks used for i-FIT = 9

Monoisotopic Mass, Even Electron Ions
425 formula(e) evaluated with 1 results within limits (all results (up to 1000) for each mass)
Elements Used:
C: 1-100 H: 0-100 N: 0-5 O: 0-16

24-May-2022 2::7::0 LCT Premier EQ45_desrat67-2 25 (0.663)
1: TOF MS ES+ 8.03e+002

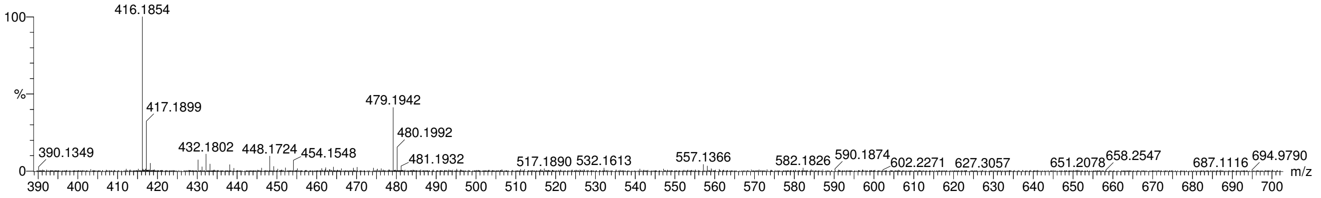

	Mass	Calc. Mass	mDa	PPM	DBE	i-FIT	i-FIT (Norm)	Formula
Minimum:					-1.5			
Maximum:			5.0	5.0	100.0			
	416.1854	416.1862	-0.8	-1.9	14.5	198.6	0.0	C26 H26 N O4

15j ^{1}H NMR $CDCl_3$ 500MHz

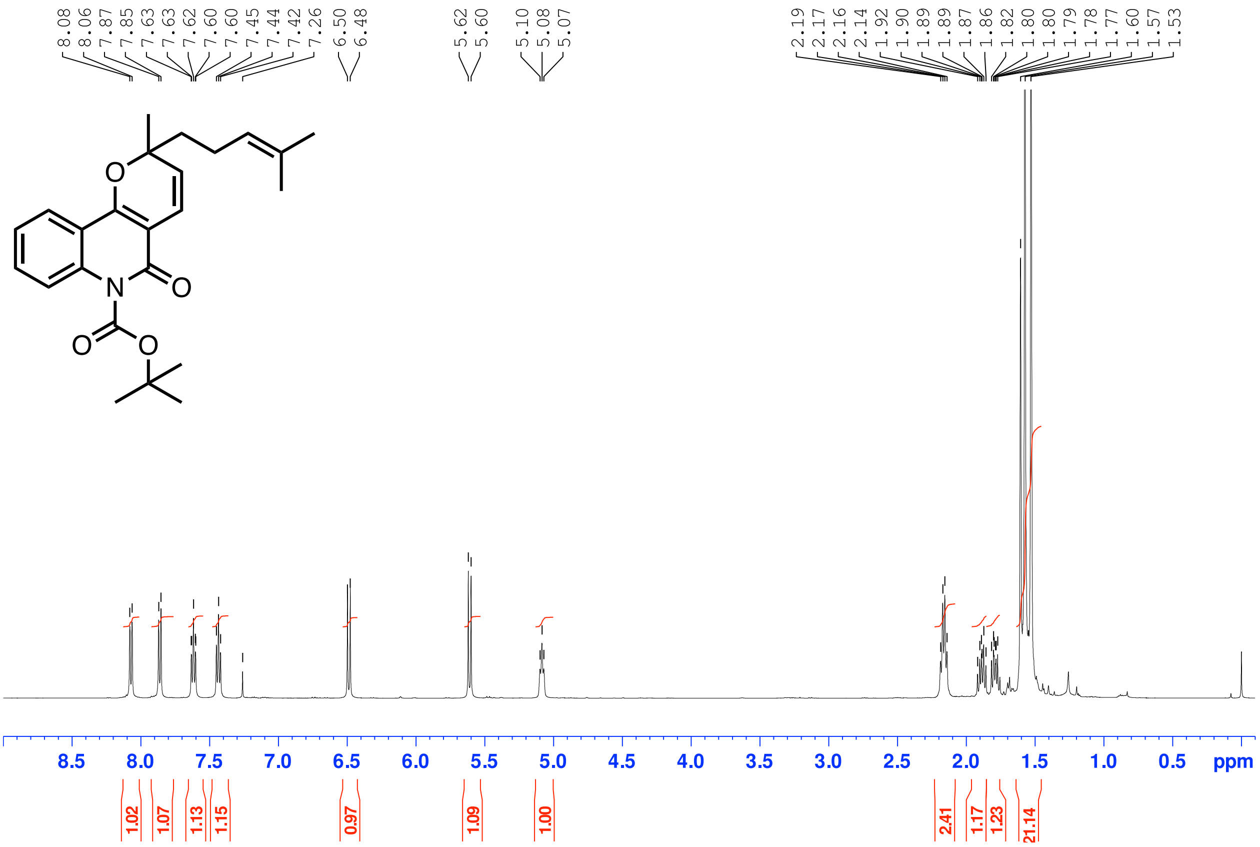

15j ^{13}C NMR $CDCl_3$ 125MHz

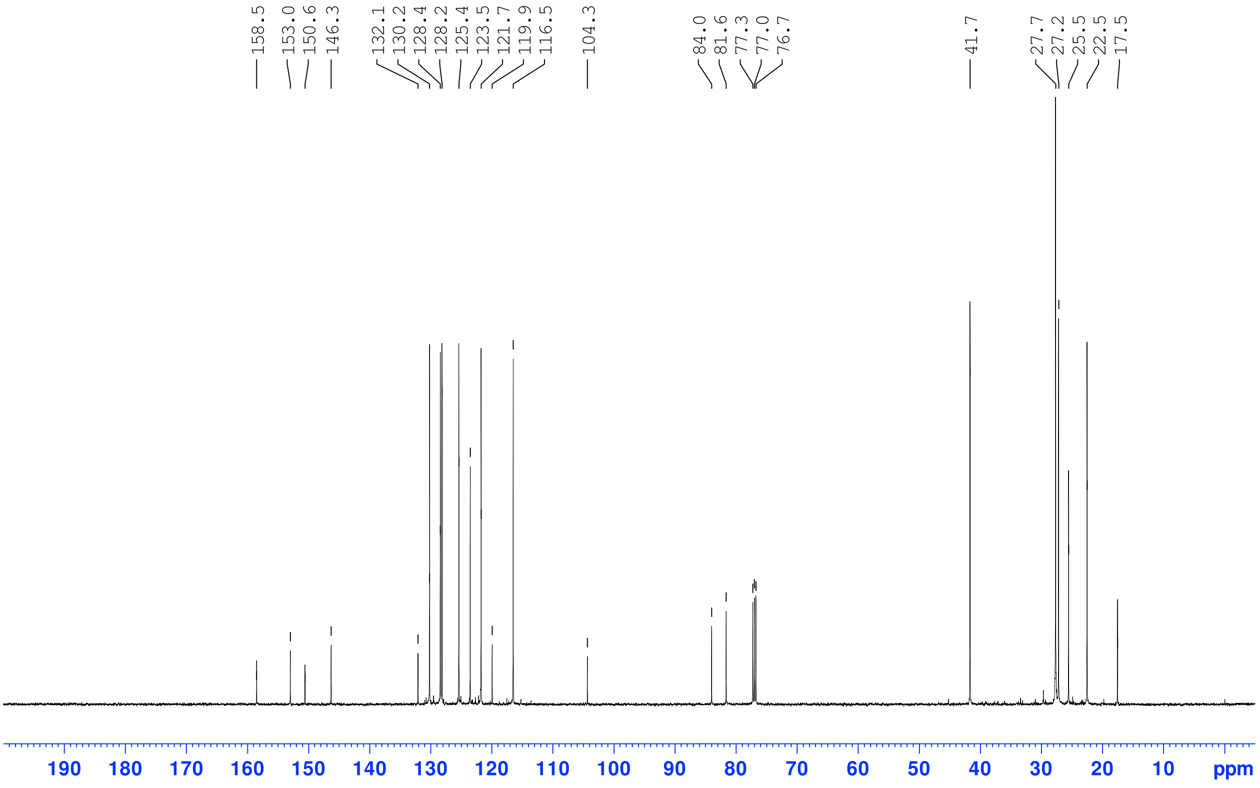

15j HRMS (ESI+)

Single Mass Analysis
Tolerance = 5.0 PPM / DBE: min = -1.5, max = 100.0
Element prediction: Off
Number of isotope peaks used for i-FIT = 9

Monoisotopic Mass, Even Electron Ions
685 formula(e) evaluated with 3 results within limits (all results (up to 1000) for each mass)
Elements Used:
C: 1-100 H: 0-100 N: 0-10 O: 0-16

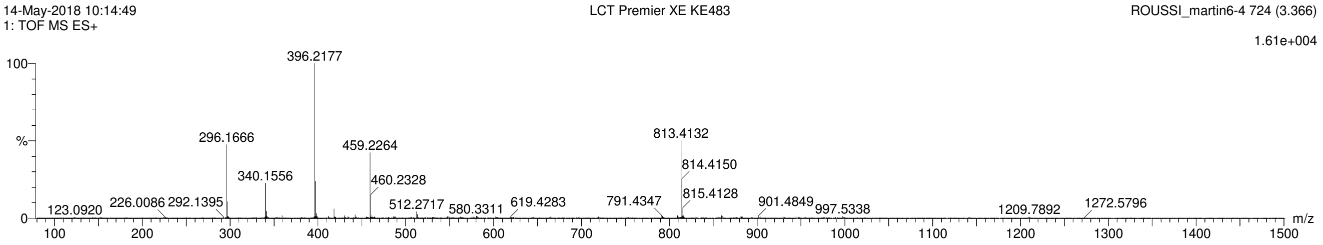

Minimum:				-1.5			
Maximum:		5.0	5.0	100.0			

Mass	Calc. Mass	mDa	PPM	DBE	i-FIT	i-FIT (Norm)	Formula
396.2177	396.2175	0.2	0.5	10.5	365.6	0.1	C24 H30 N O4
	396.2188	-1.1	-2.8	15.5	367.8	2.3	C25 H26 N5
	396.2166	1.1	2.8	-1.5	374.2	8.7	C8 H30 N9 O9

15k ^{1}H NMR $CDCl_3$ 500MHz

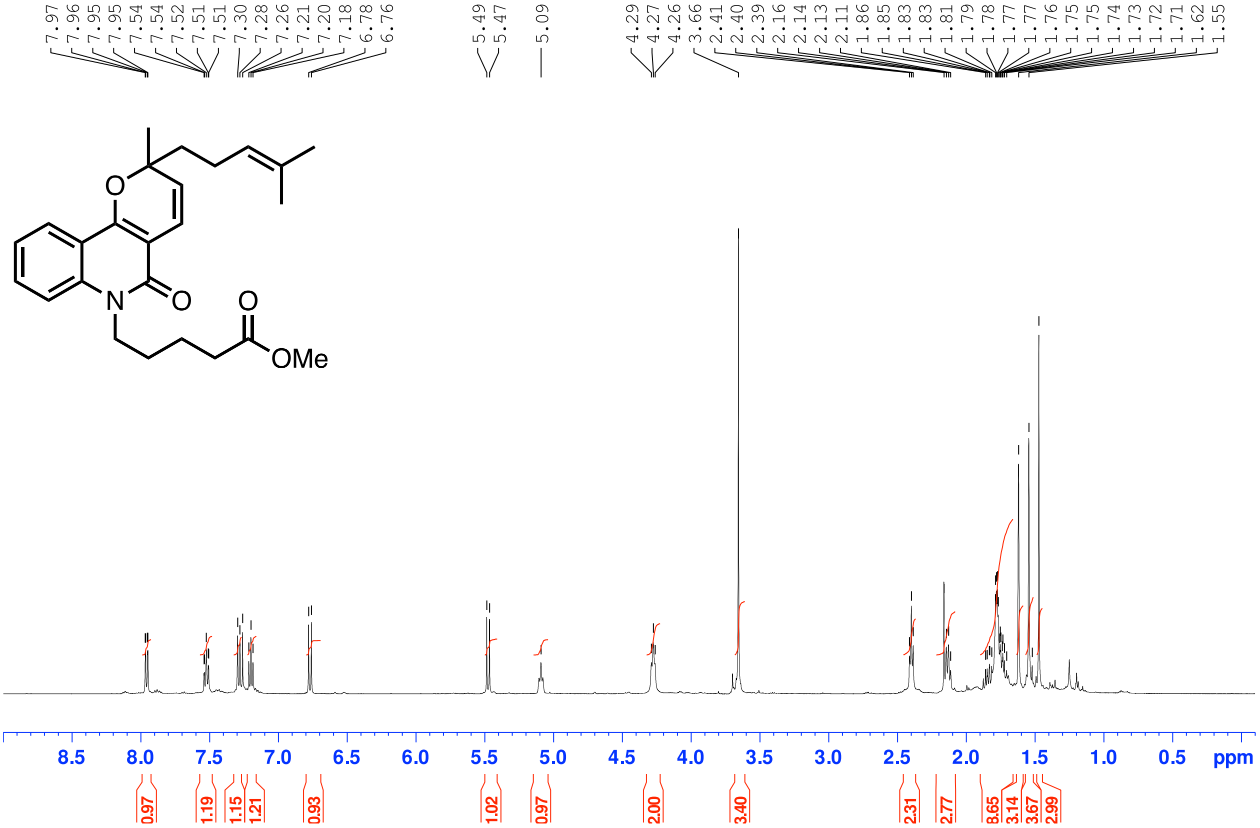

15k ^{13}C NMR $CDCl_3$ 125MHz

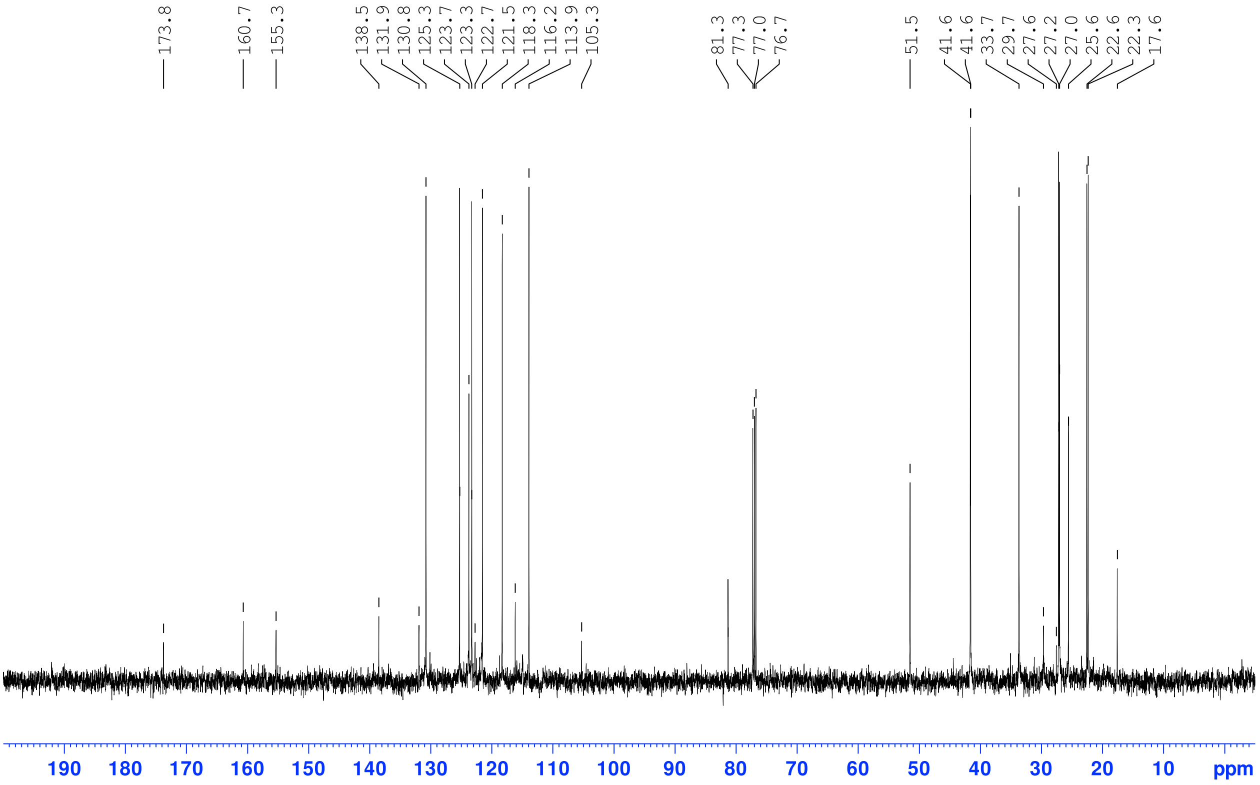

15k HRMS (ESI+)

Single Mass Analysis
Tolerance = 20.0 mDa / DBE: min = -1.5, max = 100.0
Element prediction: Off
Number of isotope peaks used for i-FIT = 9

Monoisotopic Mass, Even Electron Ions
70 formula(e) evaluated with 0 results within limits (all results (up to 1000) for each mass)
Elements Used:
C: 0-50 H: 0-120 O: 0-15

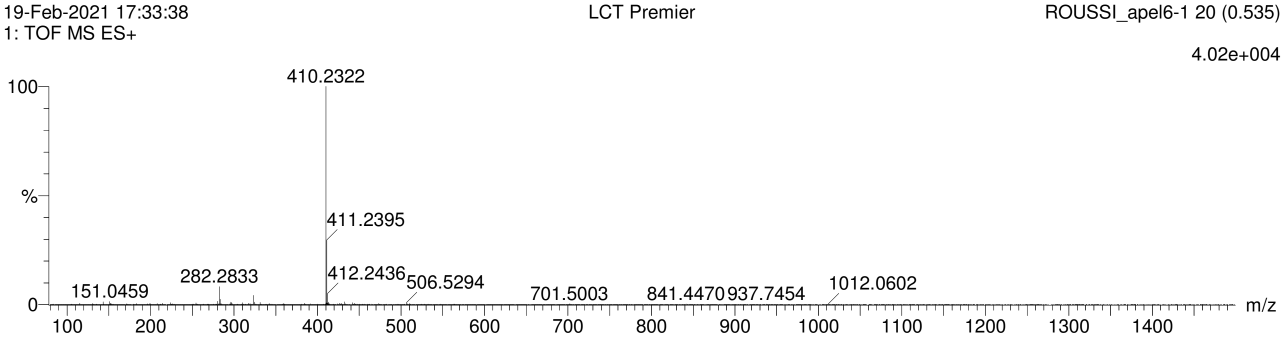


```
Minimum:                              -1.5
Maximum:              20.0      20.0      100.0

Mass       Calc. Mass    mDa     PPM     DBE     i-FIT     i-FIT (Norm) Formula

410.2322   ---
```

15l ^{1}H NMR $CDCl_3$ 500MHz

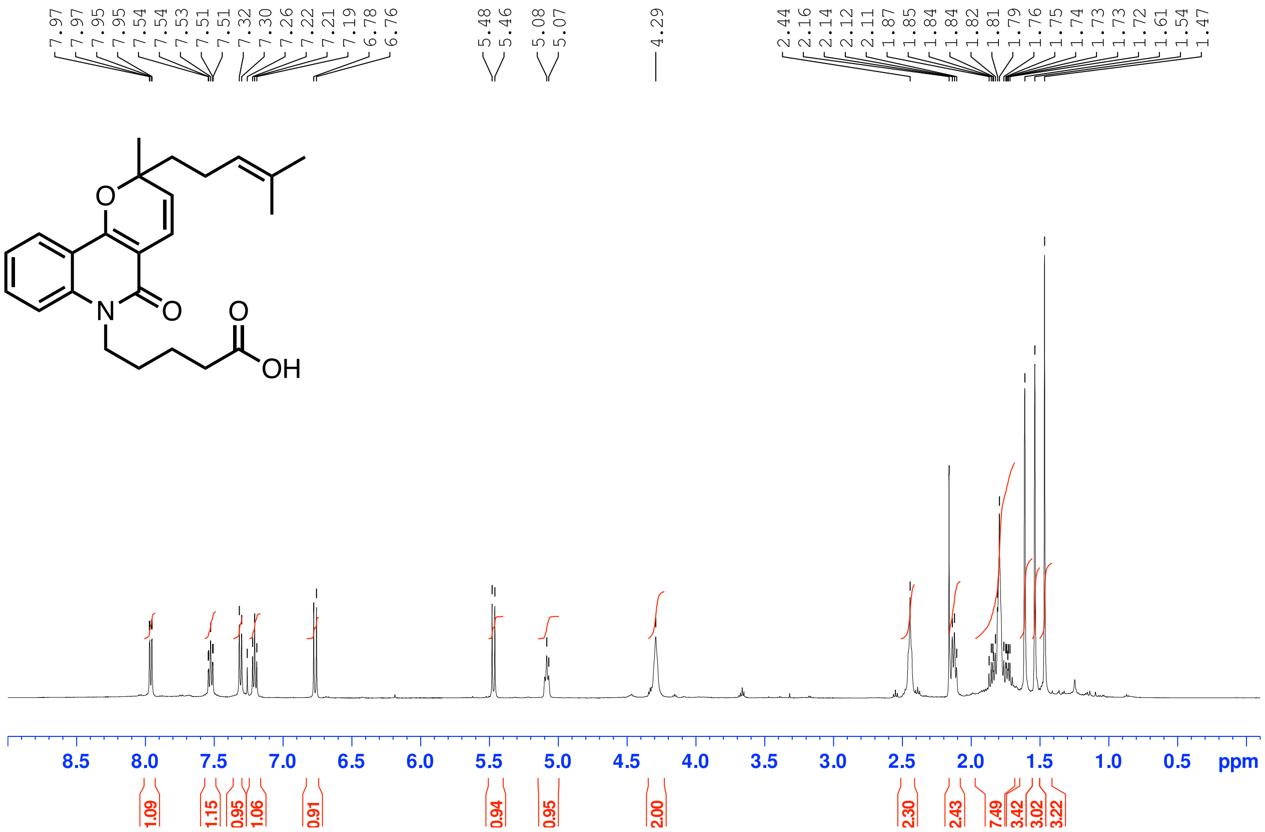

15l ^{13}C NMR $CDCl_3$ 125MHz

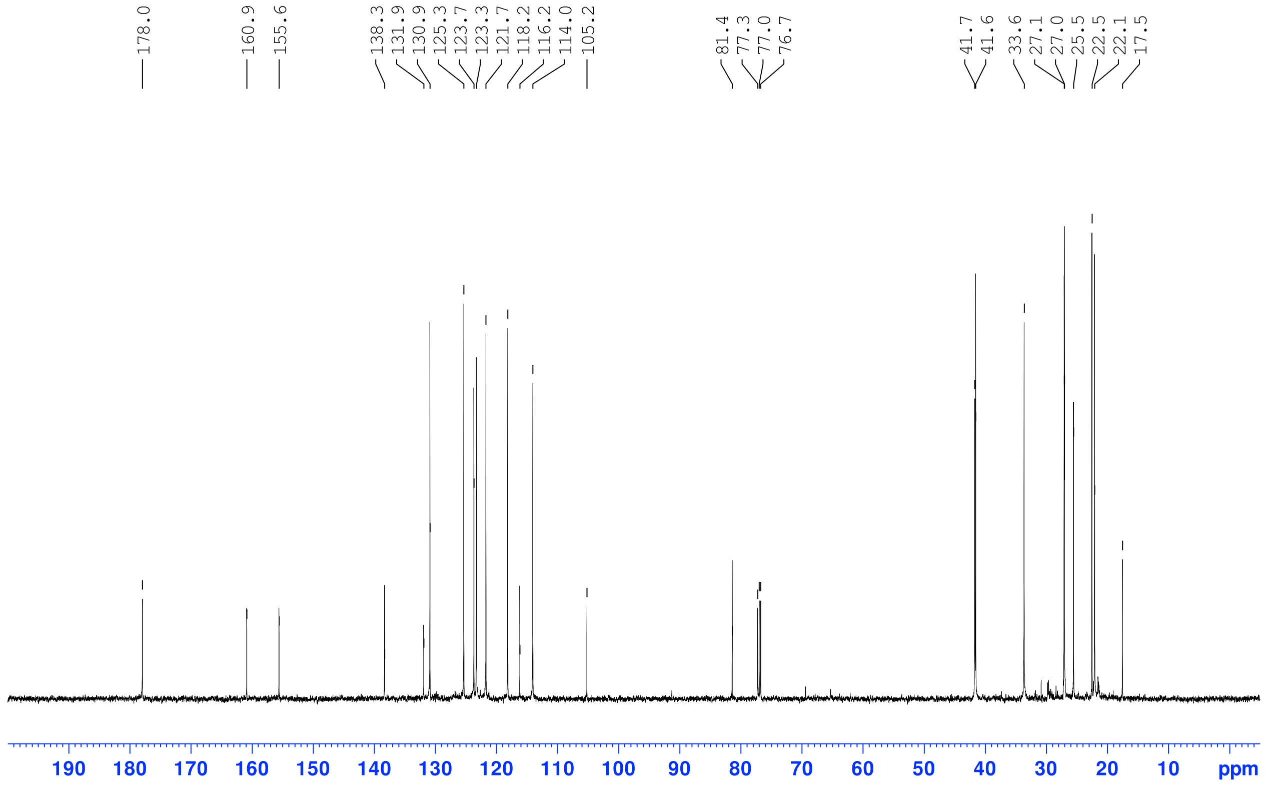

15l HRMS (ESI+)

Single Mass Analysis
Tolerance = 20.0 mDa / DBE: min = -1.5, max = 100.0
Element prediction: Off
Number of isotope peaks used for i-FIT = 9

Monoisotopic Mass, Even Electron Ions
70 formula(e) evaluated with 0 results within limits (all results (up to 1000) for each mass)
Elements Used:
C: 0-50 H: 0-120 O: 0-15

19-Feb-2021 17:42:38 LCT Premier ROUSSI_apel7-1 21 (0.571)
1: TOF MS ES+ 1.95e+004

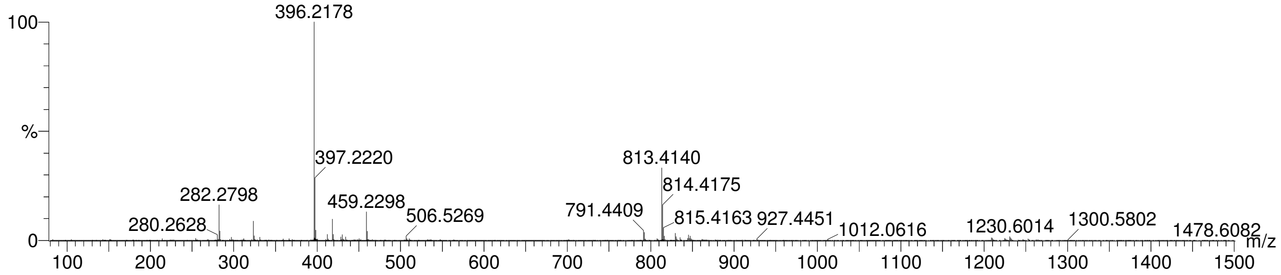


```
Minimum:                                      -1.5
Maximum:                  20.0      20.0      100.0

Mass        Calc. Mass    mDa       PPM       DBE       i-FIT       i-FIT (Norm) Formula

396.2178    ---
```

16a ^{1}H NMR $CDCl_3$ 500MHz

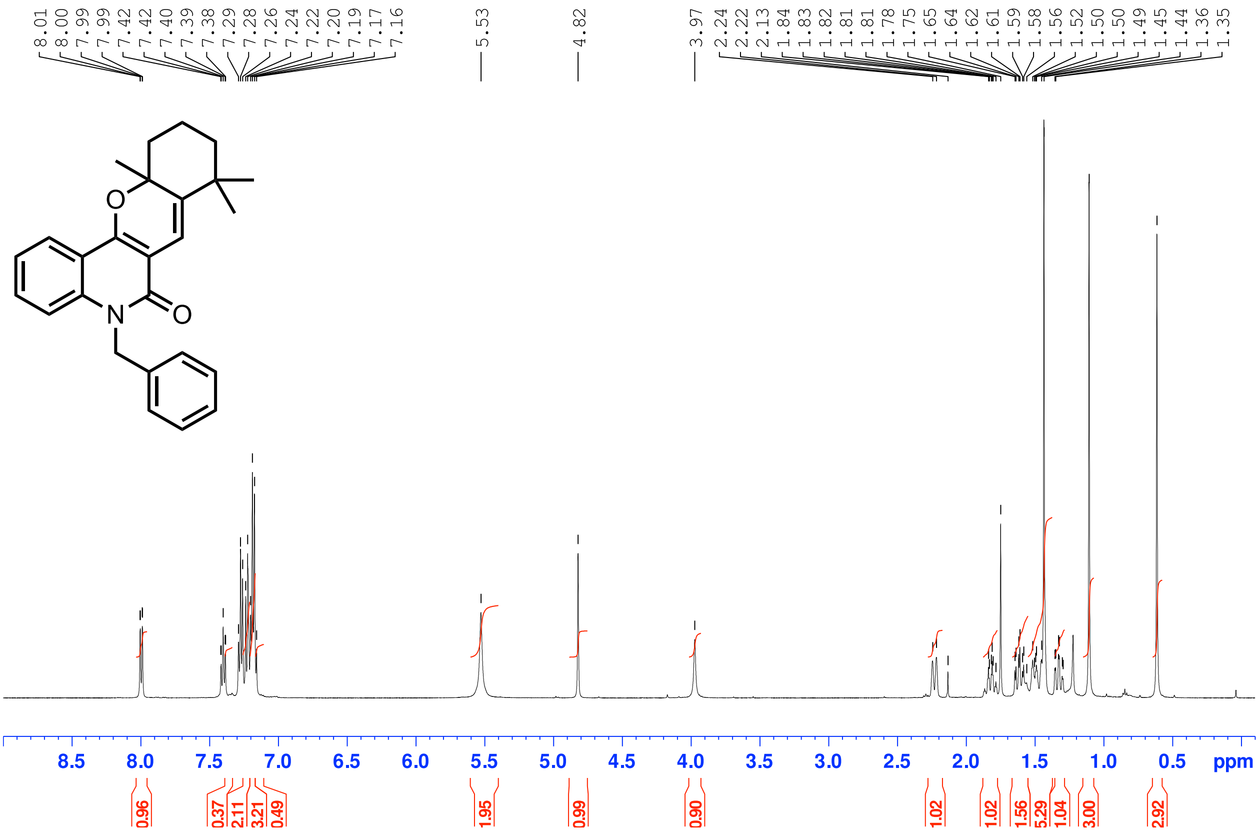

16a ^{13}C NMR $CDCl_3$ 125MHz

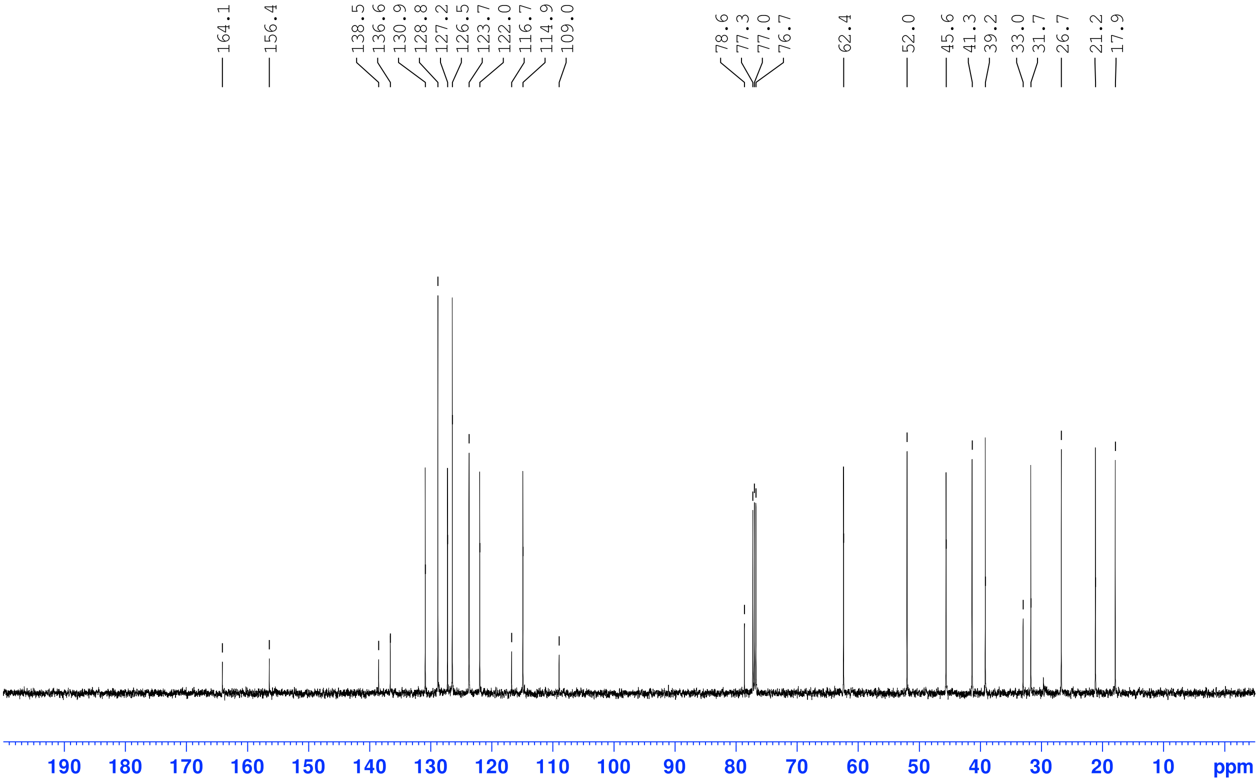

16a HRMS (ESI+)

Single Mass Analysis
Tolerance = 5.0 PPM / DBE: min = -1.5, max = 100.0
Element prediction: Off
Number of isotope peaks used for i-FIT = 9

Monoisotopic Mass, Even Electron Ions
652 formula(e) evaluated with 3 results within limits (all results (up to 1000) for each mass)
Elements Used:
C: 1-100 H: 0-100 N: 0-10 O: 0-16

19-Feb-2021 17:45:39 LCT Premier ROUSSI_apel7-2 17 (0.481)
1: TOF MS ES+

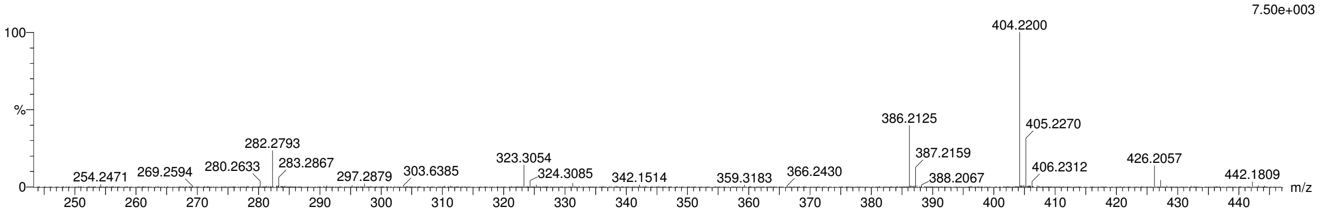

Minimum:				-1.5			
Maximum:		5.0	5.0	100.0			
Mass	Calc. Mass	mDa	PPM	DBE	i-FIT	i-FIT (Norm)	Formula
386.2125	386.2120	0.5	1.3	13.5	212.4	0.0	C26 H28 N O2
	386.2112	1.3	3.4	1.5	220.5	8.0	C10 H28 N9 O7
	386.2139	-1.4	-3.6	0.5	218.7	6.3	C14 H32 N3 O9
